# Balancing Processability and Performance: Benzoxazole Thermosets with Ultra-Low Dielectric Constants and High Thermal Stability

**DOI:** 10.3390/polym18111409

**Published:** 2026-06-05

**Authors:** Yuchen Ge, Jiaxiong Tian, Qixin Zhuang, Xiaoyun Liu

**Affiliations:** Key Laboratory of Specially Functional Polymeric Materials and Related Technology (Ministry of Education), East China University of Science and Technology, Shanghai 200237, China; 17863719189@163.com (Y.G.); qxzhuang@ecust.edu.cn (Q.Z.)

**Keywords:** polybenzoxazoles, thermosets, processability, dielectric constants, thermal stability

## Abstract

Polybenzoxazoles are promising high-performance materials for thermally stable dielectric components, microelectronic insulating layers, and aerospace-related applications owing to their exceptional thermal stability and mechanical properties; however, their poor solubility, high processing temperatures, and limited processability still restrict practical fabrication. This study presents the design and synthesis of two series of thermosetting benzoxazole monomers to address these limitations. These monomers incorporate cross-linkable arylethynyl and arylonitrile terminal groups, combined with either symmetric hexafluoroisopropylidene-bridged or asymmetric mono-benzoxazole architectures. The structure–property relationships governing solubility, curing behaviour, thermal stability, and dielectric properties are systematically investigated. The results show that incorporating hexafluoroisopropylidene units significantly enhances solubility and reduces dielectric constants, whereas nitrile-terminated systems exhibit superior thermal stability compared with their alkyne-terminated counterparts. Notably, the optimized asymmetric polybenzoxazole achieved a temperature at 5% mass loss of 602.2 °C, while the optimized symmetric polybenzoxazole exhibited an ultra-low dielectric constant of 1.83 at a frequency of 1 MHz. This work demonstrates a viable molecular design strategy for balancing solution processability, thermal stability, and dielectric performance in advanced polybenzoxazole thermosets.

## 1. Introduction

Polybenzoxazoles (PBOs) have attracted significant attention as high-performance thermosetting polymers due to their outstanding thermal stability, mechanical strength, chemical inertness, and inherent flame retardancy [1,2,3,4]. These properties make PBO suitable for demanding applications in aerospace, microelectronics, and high-temperature structural components. However, the rigid and highly conjugated molecular backbones of conventional PBO result in poor solubility, excessively high melting temperatures, and limited processability, which severely restrict their broader application [5,6]. As a result, conventional PBO processing has been largely limited to fibres and films [7,8].

The introduction of flexible groups into the molecular structure represents a common strategy to enhance solubility, as this approach increases free volume and facilitates chain mobility [9,10,11]. For example, Miyazaki et al. [12] incorporated hexafluoroisopropylidene groups into the benzoxazole framework, which significantly improved solubility and enabled dissolution in various common organic solvents. Furthermore, copolymerisation with resins that exhibit superior processability provides another established approach [13,14]. For instance, Xiao et al. [15] synthesised soluble SPBO-PI resins via copolymerisation of benzoxazole-containing diamines with 6FDA, yielding polymers soluble in toluene, γ-butyrolactone, DMSO, dichloromethane, and chloroform. However, improved solubility often reduces the efficiency of molecular chain packing, which can decrease thermal performance [16,17,18]. This trade-off remains a central challenge in the field.

The poor solubility of conventional polybenzoxazole-related systems is generally associated with their rigid aromatic backbones, strong intermolecular interactions, and limited chain mobility, while high molecular weight may further aggravate this issue in polymeric precursors [19,20,21,22]. To overcome the limitations of traditional PBO systems, our group previously proposed thermosetting PBO systems based on low-molecular-weight monomers. This approach introduces cross-linkable arylethynyl and cyano groups into the benzoxazole structure, which form cross-linked polybenzoxazole networks upon high-temperature curing. This strategy improves solubility and processability while retaining the intrinsic thermal stability and dielectric properties of PBO materials.

During thermal curing, arylethynyl groups form aromatic cross-linked structures that support the thermal stability of the resulting networks [23,24,25,26,27,28]. Chen et al. [29] reported that these groups cyclise to form structures such as naphthalene dimers and benzene trimers, which enhance thermal stability. In addition, cyano groups undergo thermal polycondensation at elevated temperatures to form triazine rings, further improving thermal resistance [30,31,32,33,34]. For example, Chen et al. [35] showed that introducing cyano groups into the side chains of poly(arylene ether nitrile) effectively maintains high thermal resistance.

Although the incorporation of hexafluoroisopropylidene (6F) units improves processability and reduces dielectric constants by increasing free volume and lowering molecular polarizability, it can reduce thermal stability because of decreased molecular packing efficiency [36,37]. Alternative structural strategies, such as constructing asymmetric mono-benzoxazole units [38,39], have therefore been explored to achieve a better balance between processability and thermal performance. In addition, arylethynyl and cyano groups have been reported as thermally reactive units for constructing crosslinked aromatic or nitrogen-containing heterocyclic networks [23,24,25,26,27,28,29,30,31,32,33,34,35]. However, direct comparisons of different backbone architectures and terminal curing groups within a unified thermosetting benzoxazole platform remain limited, especially with respect to their combined influence on processability, curing kinetics, dielectric properties, and thermal stability.

In this study, two series of thermosetting benzoxazole monomers were designed and synthesized to address this issue. The S-series consists of symmetric hexafluoroisopropylidene-bridged benzoxazole monomers, whereas the AS-series consists of asymmetric mono-benzoxazole monomers. Arylethynyl and cyano groups were introduced as terminal crosslinking groups to regulate the curing behaviour and network formation of these monomers. The solubility, rheological behaviour, non-isothermal curing kinetics, thermal stability, and dielectric properties of the monomers and their cured networks were systematically investigated. By comparing different backbone architectures and terminal reactive groups within the same thermosetting benzoxazole platform, this work aims to clarify the structure–property relationships governing processability, curing behaviour, thermal stability, and dielectric performance, thereby providing a molecular design strategy for thermosetting polybenzoxazoles with balanced processability, ultra-low dielectric constants, and high thermal stability.

## 2. Materials and Methods

### 2.1. Materials

3-Ethynylbenzaldehyde, 4-ethynylbenzaldehyde, p-cyanobenzaldehyde, m-cyanobenzaldehyde, and 2,3-dichloro-5,6-dicyano-1,4-benzoquinone were purchased from Macklin Biochemical Technology Co., Ltd. (Shanghai, China). 4-Cyano-2-fluorobenzaldehyde and 4- cyano-3-fluorobenzaldehyde were purchased from Bide Pharmatech Co., Ltd. (Shanghai, China). 2-Amino-4-bromophenol, 4-bromo-2-fluorobenzaldehyde, 5-bromo-2-hydroxybenzaldehyde, 4-bromobenzaldehyde, 4-bromo-2-hydroxybenzaldehyde, 4-bromo-3-fluorobenzaldehyde, bis(triphenylphosphine) palladium (II) dichloride, cuprous iodide, anhydrous magnesium sulfate and trimethylsilyl acetylene, 2,2-bis(3-amino-4-hydroxyphenyl) hexafluoropropane, absolute ethanol, sodium bicarbonate, and 1,4-dioxane were purchased from Aladdin Biochemical Technology Co., Ltd. (Shanghai, China). All reagents were used as received unless otherwise stated.

### 2.2. Synthesis

The detailed synthetic routes are shown in Schemes S1–S4 in the Supplementary Material. Briefly, the S-series benzoxazole monomers were synthesized through the condensation of aldehyde precursors with 2,2-bis(3-amino-4-hydroxyphenyl)hexafluoropropane, followed by oxidative cyclization using 2,3-Dichloro-5,6-dicyano-1,4-benzoquinone (DDQ). The AS-series monomers were prepared through the formation of brominated benzoxazole intermediates, Sonogashira coupling with trimethylsilylacetylene, and subsequent deprotection of the TMS groups. Detailed experimental procedures, purification methods, yields, and structural characterization data are provided in the Supplementary Material.

The structures of the eleven synthesised thermosetting benzoxazoles are shown in Figure 1. The compounds S-1, S-2, S-3, S-4, S-5, and S-6 constitute the S-series and exhibit symmetric molecular structures. AS-1, AS-2, AS-3, AS-4, and AS-5 form the AS-series with asymmetric molecular structures. Detailed synthetic procedures are provided in the Supplementary Material (Schemes S1–S4). FTIR spectra (Figures S1–S11) and 1H-NMR spectra results (Figures S12–S22) confirm the successful synthesis of these thermosetting benzoxazoles.

### 2.3. Curing of Benzoxazole Monomers

The thermosetting benzoxazole monomers were ground in an agate mortar to obtain fine powders without perceptible grit. The powders were then packed into a custom mould with an inner diameter of 10 mm and compacted under a pressure of 2 MPa using a hydraulic press to produce disc-shaped pellets with a diameter of 10 mm and a thickness of 1 mm. These pellets were subsequently cured in a tube furnace under a nitrogen atmosphere at a heating rate of 5 °C/min according to optimized stepwise curing protocols. The final curing temperature was 350 °C for S-1, S-2, S-3, S-4, S-5, S-6, and AS-1, and 380 °C for AS-2, AS-3, AS-4, and AS-5. Detailed curing schedules for each sample are provided in the Supplementary Material.

### 2.4. Methods

The solubility of the benzoxazole monomers was evaluated by adding a fixed amount of each monomer into selected organic solvents at room temperature, followed by stirring until a visually stable state was obtained. The solubility was classified as soluble (+), partially soluble (±), or insoluble (−) according to the clarity of the solution and the presence of residual solid. Proton Nuclear Magnetic Resonance (^1^HNMR) spectra were recorded on a Bruker AVANCE III 400 MHz spectrometer (Bruker, Billerica, MA, USA) using tetramethylsilane as the internal standard and deuterated dimethyl sulfoxide (DMSO-d6) as the solvent. Fourier transform infrared (FTIR) spectra of the benzoxazole monomers and cured products were obtained using a Nicolet 5700 FTIR spectrometer (Thermo Electron Corporation, Waltham, MA, USA) at room temperature via the KBr pellet method over the range of 4000–400 cm^−1^ with a resolution of 4 cm^−1^. Differential scanning calorimetry (DSC) measurements were carried out on a Diamond DSC instrument (PerkinElmer, Shelton, CT, USA) under a nitrogen atmosphere. The benzoxazole monomers were scanned from 30 to 350 °C at heating rates of 5, 10, 15, and 20 °C min^−1^, whereas the cured products were tested at a heating rate of 10 °C min^−1^ using the same instrument. Thermogravimetric analysis (TGA) was performed on an STA200 thermal analyser (HITACHI, Tokyo, Japan) from 30 to 800 °C at a heating rate of 10 °C min^−1^ under a nitrogen atmosphere, with a sample mass of 3–8 mg. The dielectric constant and dielectric loss of the cured products were measured using a Concept 40 broadband dielectric spectrometer (Novocontrol, Montabaur, Germany) over a frequency range of 10^2^–10^6^ Hz. The samples were prepared as circular discs with a diameter of 20 mm and a thickness of 1 mm. Rheological measurements were conducted on a Thermo Haake MARS III rotational rheometer (Thermo Fisher, Waltham, MA, USA) over a temperature range of 25–400 °C at a heating rate of 5 °C min^−1^.

## 3. Results and Discussion

### 3.1. Processability of Thermosetting Benzoxazoles

**Figure 2:**
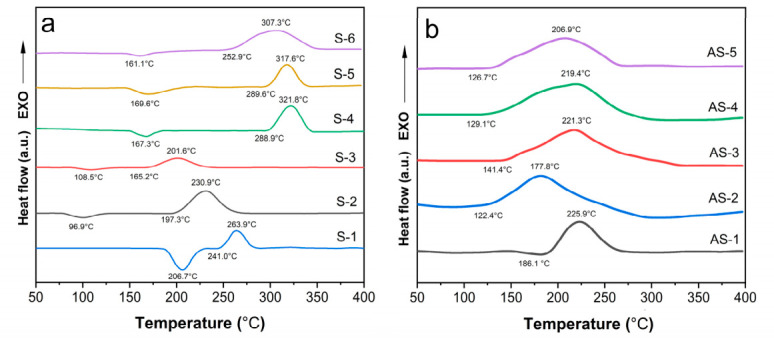
DSC curves of (**a**) S-series monomers; and (**b**) AS-series monomers.

Figure 2 shows the melting and curing behavior of all monomers. The melting point (T_m_), Initial curing temperature (T_i_) and peak curing temperatures (T_p_) can be obtained from Figure 2.

As-series benzoxazole monomers do not exhibit a distinct melting point, or their melting point is close to the curing temperature (Figure 2). Since these thermosetting benzoxazole monomers lack a well-defined melt-processing window and undergo thermally induced curing at elevated temperatures, conventional melt processing before network formation is unsuitable. Therefore, solubility and solution processability are critical for evaluating their applicability in film casting, coating, impregnation, and composite fabrication. Accordingly, their solubility serves as a preliminary indicator of monomer processability. The solubility of eleven monomers was examined in commonly used solvents, and the results are summarised in Table 1.

As shown in Table 1, none of the eleven monomers is soluble in ethanol. Overall, the S-series exhibits markedly better solubility than the AS-series. This difference arises from replacing the single phenyl bridging unit in the benzoxazole framework with a hexafluoroisopropylidene moiety, which disrupts conjugation and reduces molecular rigidity. Compared with conventional PBO, the AS-series may also exhibit improved solubility due to the lower molecular weight associated with the small-molecule design. In addition, substituents on the benzoxazole unit break molecular symmetry, which reduces packing efficiency and rigidity. Variations among these substituents exert only a minor influence on solubility, as shown in Table 1.

**Figure 3:**
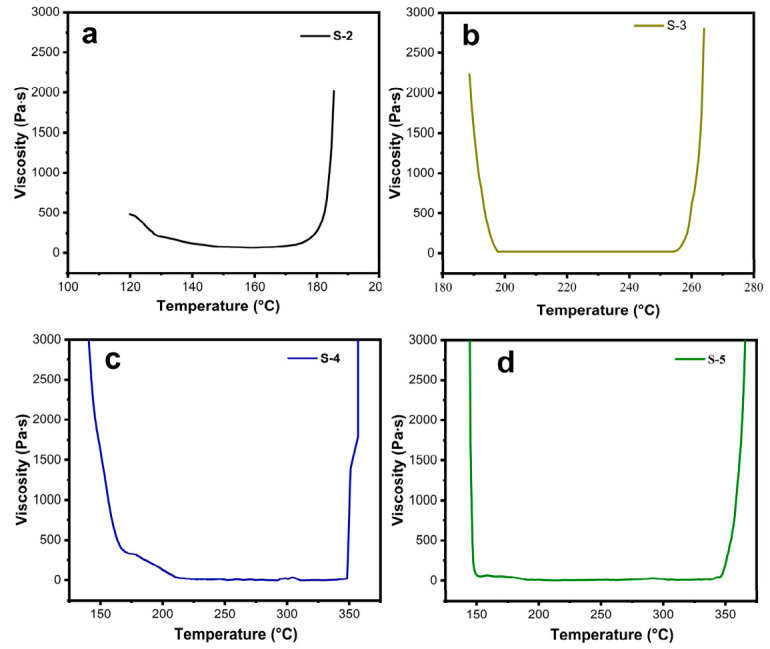
Viscosity vs. temperature curve: (**a**) S-2, (**b**) S-3, (**c**) S-4, (**d**) S-5.

The viscosity vs. temperature curve of the benzoxazole monomers was investigated using a rotational rheometer to assess processability (Figure 3). As temperature increases, all monomers exhibit a characteristic three-stage viscosity profile: an initial decrease due to melt flow, a low-viscosity plateau defining the processing window, and a sharp increase associated with curing onset.

In addition, although S-1 and S-6 monomers have melting points, their melt viscosity is too high and can only be processed by solution method. Therefore, the viscosity data of S-1 and S-6 are not provided in Figure 3. Table 2 shows the melting points and curing behavior parameters of the S-series monomers. Since the AS-series monomers have no melting point and can only be processed using solution method, they are not listed in Table 2.

Among the benzoxazole arylethynyl monomers, S-2 exhibits a viscosity of 101.2 Pa·s and a processing window of 132–178 °C, which indicates a relatively narrow temperature range for melt processing. In contrast, S-1 does not display a well-defined flow profile. This behaviour is attributed to the para-positioned ethynyl group in S-1, which results in a highly regular molecular architecture. Such regularity promotes efficient packing upon melting, restricts segmental mobility, and leads to high viscosity. In comparison, the meta-substituted ethynyl group in S-2 disrupts symmetry, which facilitates melt flow and improves processability.

For the benzoxazole arylonitrile monomers, S-3, S-4, and S-5 exhibit pronounced melt flow and substantially broader processing windows. Specifically, S-3 shows a viscosity of 20.6 Pa·s with a processing window of 194–261 °C. S-4 and S-5 exhibit viscosities of 21.4 and 22.7 Pa·s, with processing windows of 193–348 °C and 148–350 °C, respectively. These values indicate comparable viscosities among the arylonitrile monomers, all of which are significantly lower than those of the arylethynyl counterparts. This behaviour is plausibly related to the higher curing activation energy of nitrile groups relative to ethynyl groups. At lower temperatures, nitrile-containing systems are less prone to premature crosslinking, which allows them to maintain low viscosity and provides a broader processing window. In contrast, S-6 exhibits poor flow behaviour. This behaviour may arise from the proximity of the fluorinated substituent to the oxazole ring, which enhances conjugation with adjacent phenyl and oxazole units and restricts molecular mobility. Strong intermolecular interactions involving fluorinated groups may further limit thermal motion. Consequently, hot-press moulding may represent a more suitable processing route for S-6. For S-3, the highly symmetric molecular structure promotes efficient packing and intermolecular interactions, which result in a slightly narrower processing window than those of the other nitrile monomers. The broader processing window of S-5 compared with S-4 likely reflects the dominant influence of pendant substituents, which introduce steric hindrance, reduce structural regularity, and increase free volume, thereby facilitating molecular motion.

### 3.2. Curing Properties of Monomers

As shown in Figure 2, all monomers exhibit distinct exothermic events in the high-temperature region, which confirms thermally induced curing during heating. However, pronounced differences appear in the onset temperature, peak position, and peak profile of the exotherms, which indicates that the curing response is highly sensitive to both terminal group chemistry and overall molecular architecture. In general, within the symmetric diphenyl series, the alkyne-terminated monomers cure at substantially lower temperatures than the nitrile-terminated analogues. The asymmetric monophenyl series also shows exothermic processes within a lower temperature window. These observations indicate that curing behaviour arises from the combined effects of intrinsic end-group reactivity and the packing or organisation state of the monomers. Therefore, non-isothermal DSC scans were performed to extract T_p_ for kinetic analysis using Kissinger and Ozawa methods [40,41]. In the Kissinger method, the apparent activation energy (Ea) is obtained from the linear relationship between ln(β/T_p_^2^) and 1/T_p_:

$$\ln\left(\frac{\beta }{{T_{p}}^{2}}\right)=\ln\left(\frac{AR}{E_{a}}\right)-\frac{E_{a}}{\left(RT_{p}\right)}$$

where β is the heating rate, T_p_ is the exothermic peak temperature in Kelvin, A is the pre-exponential factor, and R is the gas constant. Thus, Ea can be calculated from the slope of the ln(β/T_p_^2^) versus 1/T_p_ plot. The Ozawa method was also used for comparison, according to the following equation:

$$\ln\beta =C-1.052\frac{E_{a}}{RT_{p}}$$

where C is a constant. In this method, Ea is calculated from the slope of the log β versus 1/T_p_ plot. The use of both methods allows the curing activation energies to be cross-checked using two independent non-isothermal kinetic approaches. The complete thermograms are provided in the Supplementary Material (Figures S23–S33). The complete kinetic analysis using Kissinger and Ozawa methods is provided in the Supplementary Material (Figures S34–S44, Tables S2 and S3).

**Figure 4:**
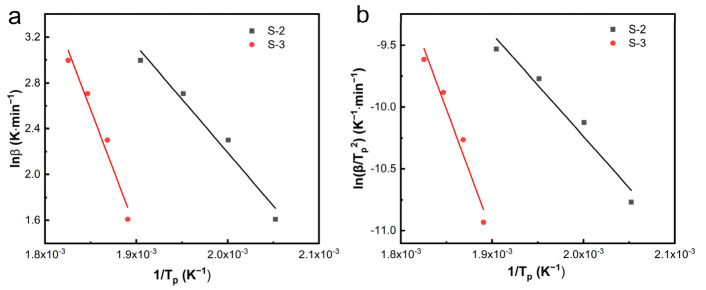
(**a**) Kissinger plots; (**b**) Ozawa plots of monomers.

As shown in Figures S23–S33, with increasing heating rate, T_p_ shifts systematically towards higher temperature for all samples. This trend is characteristic of a kinetically controlled curing process and supports the use of peak-based methods. Accordingly, the apparent activation energy (Ea) was calculated using the Kissinger and Ozawa approaches. Representative linear fits for S-2 and S-3 (Figure 4) show good linear correlations, which confirm the applicability of these methods for comparing curing kinetics across different monomers. The calculated Ea values are summarised in Table 3. The close agreement between the two methods supports the reliability of the extracted kinetic parameters.

As summarised in Table 3, the nature of the terminal group is the dominant factor controlling the curing kinetics. In the symmetric diphenyl series, the average Ea values of the alkyne-containing monomers S-2 and S-1 are 71.25 and 72.10 kJ mol^−1^, respectively, which are markedly lower than those of the nitrile-containing monomers S-3, S-4, and S-5 (106.1, 116.1, and 138.5 kJ mol^−1^, respectively). Although S-6 shows a lower Ea of 91.71 kJ mol^−1^, this value remains higher than those of the alkyne-based systems. This comparison indicates that, in these thermosetting benzoxazole systems, alkyne-involved curing proceeds more readily than nitrile-involved curing.

The apparent activation energy (Ea) is an important kinetic parameter for evaluating the thermal curing behaviour of thermosetting monomers. It reflects the overall energy barrier required for network formation under non-isothermal conditions and provides useful guidance for comparing curing reactivity, optimizing curing schedules, and assessing the processing window of different monomer structures. A lower Ea generally indicates that the curing reaction can proceed more readily at lower thermal input, whereas a higher Ea suggests a more restricted curing process that requires higher temperature or greater molecular mobility. This difference can be explained by the distinct network-forming pathways of the two terminal groups. The curing behaviour of the alkyne-terminated benzoxazole monomers is consistent with previous studies on arylethynyl- and phenylethynyl-terminated high-temperature thermosetting systems. In these systems, thermal curing of arylethynyl groups has been reported to involve complex reactions, including dimerization, radical chain growth, cyclization, cyclotrimerization and crosslinking, rather than a single elementary reaction pathway [28,29]. Similar DSC-based kinetic analyses using Kissinger and Ozawa methods have also been widely applied to evaluate the curing reactivity of phenylethynyl- or arylacetylene-containing resins [25,27]. Compared with the nitrile-terminated analogues in this work, the alkyne-terminated monomers exhibited lower curing peak temperatures and lower apparent activation energies, indicating higher curing reactivity of the arylethynyl end groups. This trend agrees with the reported high thermal reactivity of arylethynyl-containing thermosetting systems, although the specific curing temperature and activation energy are strongly dependent on the molecular structure and end-group concentration. In contrast, curing of nitrile-containing monomers mainly involves high-temperature cyclisation of nitrile groups to form triazine or related nitrogen-containing heterocycles [30,31,32,33,34,35]. These reactions are sensitive to steric constraints, segmental mobility, and the spatial approach of reactive groups, which requires higher thermal input. As a result, nitrile systems exhibit delayed exotherms and consistently higher Ea values. The distinct kinetic parameters and curing windows of the two series therefore arise from the difference between progressive alkyne-based crosslinking and more demanding nitrile-based cyclisation.

Within each end-group category, substituent identity and substitution pattern further influence curing behaviour. Among nitrile-terminated monomers, S-6 shows the lowest Ea, whereas S-5 exhibits the highest value. This trend indicates that substitution mode strongly affects nitrile reactivity by altering dipolar interactions, conformational freedom, and packing regularity. Because nitrile cyclisation is a geometry-sensitive process, increased rigidity, tighter packing, or limited accessibility raises the apparent kinetic barrier. A similar structure-dependent trend appears in the asymmetric monophenyl series. AS-1, AS-4, AS-5, AS-3, and AS-2 exhibit average Ea values of 54.72, 61.11, 66.06, 80.92, and 43.90 kJ mol^−1^, respectively. Curing in this series remains dominated by alkyne conversion, and the Ea values are therefore lower than those of nitrile-containing monomers.

Notably, AS-2 shows the lowest Ea, which indicates the highest curing reactivity, whereas AS-3 exhibits a significantly higher Ea. This difference suggests a strong positional dependence of the hydroxyl group. In AS-2, the hydroxyl group likely promotes local activation through conjugative effects, hydrogen bonding, and possible proton-transfer-assisted polarisation, which facilitates alkyne conversion at a lower temperature.

In contrast, AS-3 likely exhibits stronger intermolecular association and restricted conformational flexibility, which hinders effective contact between reactive sites and increases the kinetic barrier. Halogen-substituted monomers (AS-1, AS-4, and AS-5) show intermediate-to-low Ea values, which suggests that polarizable substituents disrupt excessive packing and favour alkyne-based curing.

**Figure 5:**
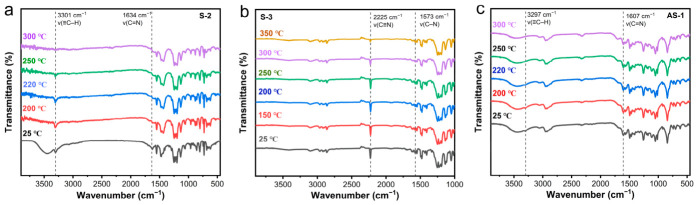
Variable-temperature FTIR spectra of (**a**) S-2; (**b**) S-3; and (**c**) AS-1 during thermal curing.

The kinetic interpretation is further supported by the in situ FTIR results shown in Figure 5. For S-2, the characteristic absorption of terminal alkyne C-H at 3301 cm^−1^ gradually weakens with increasing temperature and nearly disappears at high temperature, which indicates continuous consumption of the alkyne group during curing. In addition, absorptions in the 1400–1600 cm^−1^ region increase, which is consistent with the formation of more conjugated and aromatic crosslinked structures. AS-1 shows a similar evolution at 3297 cm^−1^, which confirms that monomers in the AS-series also cure predominantly through alkyne conversion. Combined with their relatively low Ea values, these spectral changes demonstrate that alkyne-containing systems undergo a continuous transition from end-group activation to crosslink formation within a relatively low temperature range.

In contrast, the spectral evolution of S-3 reflects the higher barrier associated with nitrile curing. The characteristic C≡N absorption at 2225 cm^−1^ remains prominent over 150–250 °C and decreases substantially only at higher temperatures, which indicates delayed nitrile conversion. At the same time, enhanced absorption around 1634 cm^−1^ corresponds to C=N stretching in triazine or related heterocycles, while changes near 1576 cm^−1^ are associated with the formation of C-N bonds. These observations indicate that nitrile groups are transformed into cyclic nitrogen-containing crosslinking structures rather than simply consumed. Because this pathway requires cooperative interaction and cyclisation of multiple nitrile groups, it is more sensitive to temperature and molecular mobility. The in situ FTIR results, therefore, provide direct spectroscopic evidence for the origin of the higher Ea values of the nitrile-terminated monomers. Overall, the DSC, kinetic analysis, and in situ FTIR results consistently show that the curing behaviour of these benzoxazole monomers is governed by the interplay between terminal-group reaction pathways and molecular structure.

Alkyne-terminated monomers undergo earlier and kinetically more accessible curing, whereas nitrile-terminated monomers require higher-temperature cooperative cyclisation to achieve network formation.

### 3.3. Thermal Stability of Polybenzoxazoles

**Figure 6:**
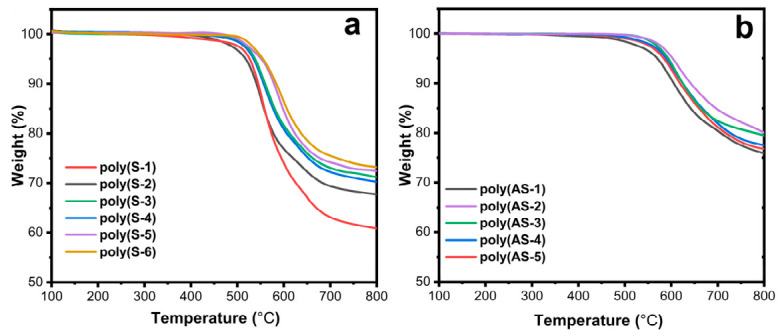
TGA curves of (**a**) S-series polybenzoxazoles; and (**b**) AS-series polybenzoxazoles.

The thermal stability of polybenzoxazoles was evaluated by TGA under nitrogen (Figure 6), and the data are summarised in Table 4. As shown in Figure 6, all polybenzoxazoles exhibit high thermal decomposition temperature at 5% mass loss (T_d5_), and high residual carbon ratio at 800 °C (Y_c,800_). This indicates that the crosslinked aromatic heterocyclic networks remain stable at elevated temperatures.

For the arylethynyl/arylnitrile-containing systems, both the type of crosslinkable group and its substitution position influence thermal resistance. Within the same functional group, para-substituted structures show slightly higher T_d5_ values than the corresponding meta-substituted analogues. For example, poly(S-1) exhibits a T_d5_ of 525.7 °C, which exceeds that of poly(S-2) (517.0 °C), while poly(S-3) shows a slightly higher T_d5_ (537.8 °C) than poly(S-4) (534.8 °C). This trend indicates that para-substitution promotes more regular and tightly packed networks, which suppress segmental motion and delay thermal decomposition.

A more pronounced difference appears between ethynyl- and nitrile–functionalised systems. Overall, nitrile-containing monomers show higher thermal stability than the corresponding ethynyl analogues. This behaviour can be attributed to the formation of thermally robust triazine or phthalocyanine-like structures during nitrile curing. In contrast, ethynyl curing, although capable of forming aromatic structures via cyclotrimerisation, may also generate less stable conjugated segments under high-temperature conditions. As a result, nitrile-derived networks exhibit greater resistance to thermal degradation. This trend is further supported by the fluorinated nitrile systems, where poly(S-5) and poly(S-6) show T_d5_ values of 550.5 and 554.1 °C, respectively, both exceeding those of poly(S-3) and poly(S-4).

Fluorine incorporation further enhances thermal stability in the nitrile-based series. Compared with the non- or less-optimised analogues, fluorinated structures exhibit improved thermal stability, which can be associated with the strong electron-withdrawing nature of fluorine and enhanced intermolecular interactions. In addition, when the fluorine substituent is positioned closer to the oxazole backbone, restricted bond rotation and increased chain rigidity further stabilise the network. Thermal stability in these systems, therefore, depends not only on crosslinking chemistry but also on electronic and steric effects.

Compared with the arylethynyl/arylnitrile systems, the second group of cured polybenzoxazoles exhibits even higher thermal stability. The lowest T_d5_ in this series, observed for poly(AS-1), reaches 569.5 °C, which is 15.4 °C higher than that of poly(S-6), the most thermally stable sample in the previous group. This result indicates that intrinsic rigidity is a key determinant of thermal stability. The poly(AS-1)-type backbone is mainly composed of benzene and oxazole rings with highly conjugated structures and minimal flexible linkages, which provides a rigid framework resistant to thermal degradation.

Within this rigid-backbone series, side-group modification further improves the thermal stability. Upon introducing fluorinated side groups, the T_d5_ values increase from 569.5 °C for poly(AS-1) to 582.9 and 588.9 °C for poly(AS-5) and poly(AS-4), respectively. This enhancement arises from stronger intermolecular interactions associated with polar C-F bonds and increased local rigidity. Replacing fluorinated substituents with hydroxyl groups leads to a further increase in thermal stability, with T_d5_ values of 593.5 °C for poly(AS-3) and 602.2 °C for poly(AS-2). The superior performance of the hydroxyl-containing monomers can be attributed to the formation of intermolecular hydrogen bonds, which are stronger and more directional than the inductive effects provided by fluorine alone. These hydrogen-bonding interactions promote tighter chain packing and improved structural regularity, thereby producing the most thermally stable networks among all samples investigated.

Taken together, the TGA results demonstrate that the thermal stability of cured polybenzoxazole is determined cooperatively by three structural factors: the nature of the cross-linkable group, the substitution pattern, and the rigidity and intermolecular interactions of the polymer backbone. Nitrile-based systems are generally more thermally stable than ethynyl-based ones, para-substitution is typically more favourable than meta-substitution, and the incorporation of rigid backbones, fluorinated groups, or especially hydroxyl groups further enhances resistance to thermal degradation. These results clearly reveal the structure–thermal stability relationship in this class of high-performance polybenzoxazole and provide useful guidance for the molecular design of thermally robust polymer systems.

### 3.4. Dielectric Properties of Polybenzoxazoles

**Figure 7:**
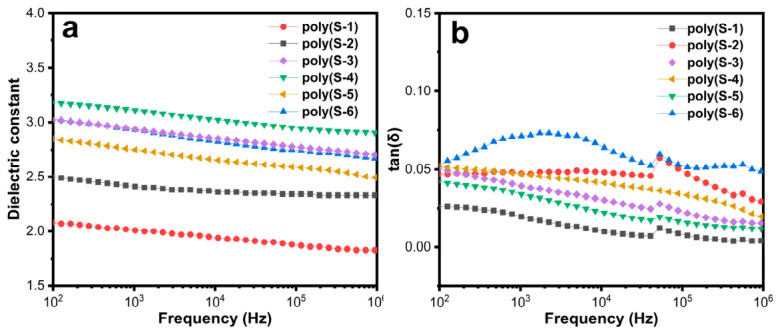
Dielectric properties of S-series polybenzoxazoles: (**a**) dielectric constant (**b**) dielectric loss.

**Figure 8:**
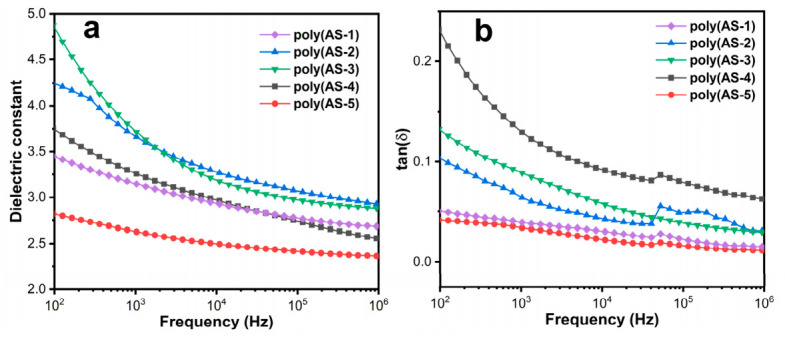
Dielectric properties of AS-series polybenzoxazoles: (**a**) dielectric constant (**b**) dielectric loss.

The dielectric properties of the prepared polybenzoxazoles were evaluated at room temperature over the frequency range of 10^2^–10^6^ Hz using a broadband dielectric spectrometer. As shown in Figure 7 and Figure 8, most samples exhibit relatively stable dielectric responses across the measured frequency range, with only minor variations in dielectric constant and dielectric loss. In particular, the S-series polybenzoxazoles show very weak frequency dependence, whereas the hydroxyl-containing AS-series samples, especially poly(AS-2) and poly(AS-3), exhibit a more obvious decrease in dielectric constant with increasing frequency, as discussed below. These results indicate that the dielectric response of the cured polybenzoxazole networks is closely related to molecular structure, polar-group content, and network mobility.

For the arylethynyl/arylnitrile-containing polybenzoxazoles, namely poly(S-1), poly(S-2), poly(S-3), poly(S-4), poly(S-5), and poly(S-6), the dielectric constants remain nearly unchanged throughout the tested frequency range. This weak frequency dependence can be understood from the viewpoint of Maxwell–Wagner interfacial polarization and Koop’s phenomenological theory [42,43]. In heterogeneous dielectric systems, interfacial polarization or space-charge accumulation usually contributes significantly to the dielectric constant at low frequencies, because dipoles and charge carriers have sufficient time to respond to the alternating electric field. With increasing frequency, these polarization processes become increasingly restricted, leading to a decrease in dielectric constant. In the present S-series polybenzoxazole networks, however, the dielectric constant decreases only slightly with frequency, indicating that interfacial polarization and mobile-charge accumulation are effectively suppressed. This behaviour can be attributed to the highly cross-linked network structure, restricted segmental mobility, low concentration of mobile charge carriers, and relatively homogeneous distribution of polarizable units. According to Koop’s phenomenological theory, such weak dielectric dispersion suggests that resistive barriers within the material effectively limit long-range charge migration, thereby maintaining stable dielectric properties over a wide frequency range. The dielectric constant follows the order poly(S-1) < poly(S-2) < poly(S-5) < poly(S-6) < poly(S-3) < poly(S-4), with poly(S-1) exhibiting the lowest value of 1.83 at 1 MHz. This behaviour can be mainly attributed to the incorporation of the hexafluoroisopropylidene unit, which lowers overall molecular polarity and simultaneously increases free volume, thereby reducing the density of polarizable groups per unit volume [36,37]. In addition, the arylethynyl-polybenzoxazoles generally show lower dielectric constants than the arylnitrile analogues, because the strongly polar nitrile groups enhance molecular polarity and intermolecular interactions, resulting in tighter chain packing and reduced free volume. By contrast, ethynyl groups can undergo thermal curing to form rigid aromatic cross-linked structures, which restricts dipolar relaxation and helps reduce dielectric polarization. A positional effect is also evident: para-substituted polybenzoxazoles consistently exhibit lower dielectric constants than their meta-substituted counterparts, suggesting that higher structural symmetry and better network regularity help suppress polarization.

For the asymmetric arylethynyl polybenzoxazoles, including poly(AS-1), poly(AS-2), poly(AS-3), poly(AS-4), and poly(AS-5), the dielectric constants are also relatively stable over the measured frequency range. However, poly(AS-2) and poly(AS-3) show a more obvious decrease with increasing frequency compared with the other AS-series samples. This behaviour is mainly attributed to the hydroxyl groups in their molecular structures. At low frequencies, the polar hydroxyl groups can contribute to orientational polarization because they have sufficient time to respond to the alternating electric field. As the frequency increases, this dipolar response becomes increasingly restricted owing to hydrogen-bonding interactions and limited segmental mobility, resulting in a gradual decrease in dielectric constant. The dielectric constant follows the order poly(AS-5) < poly(AS-4) < poly(AS-1) < poly(AS-2) < poly(AS-3), with poly(AS-5) showing the lowest value of 2.37 at 1 MHz. These results highlight the important role of side-group engineering in regulating dielectric properties. Fluorinated side groups decrease molecular polarizability and increase free volume, thereby lowering the dielectric constant. In contrast, hydroxyl groups increase molecular polarity, strengthen intermolecular interactions through hydrogen bonding, and may also increase moisture uptake, all of which contribute to higher dielectric constants. The slightly lower dielectric constant of poly(AS-2) relative to poly(AS-3) further suggests that differences in substitution position, molecular packing, and curing completeness can influence dielectric polarization.

The dielectric loss of polybenzoxazoles generally decreases with increasing frequency, although the overall variation remains limited. For the arylethynyl/arylnitrile systems, poly(S-6) exhibits the highest dielectric loss, which may be associated with its relatively strong conjugation effect that facilitates charge migration under an alternating electric field. For the para-substituted arylethynyl systems, poly(AS-4) shows comparatively higher dielectric losses; this is likely related to enhanced charge transport arising from conjugation. Apart from the above samples, most polybenzoxazoles maintain low dielectric losses, with values below 0.05 at 1 MHz, which indicates favourable low-loss characteristics for this class of materials. To further evaluate the charge-transport behaviour, the AC conductivity was calculated from the dielectric data according to the following equation:

σac=2πfε0ε’tanδ

where f is the frequency, ε0 is the vacuum permittivity, ε′ is the dielectric constant, and tan δ is the dielectric loss tangent. The AC conductivity increases gradually with increasing frequency, which is typical for insulating polymeric dielectrics under alternating electric fields. The low conductivity values over the measured frequency range further confirm the excellent electrical insulating characteristics of the cured polybenzoxazole networks. The differences in σac among different samples are consistent with the dielectric loss results and can be attributed to variations in polar-group content, cross-linking density, molecular packing, and charge-transport ability. The corresponding frequency-dependent AC conductivity curves are shown in Figure S45.

Compared with previously reported high-temperature-resistant dielectric polymers, the present polybenzoxazoles show a favourable balance between low dielectric constant, low dielectric loss, and high thermal stability. Many reported polyimides, bismaleimide-based thermosets, and related high-temperature polymers generally exhibit dielectric constants above 2.5–3.5 at 1 MHz. In contrast, poly(S-1) in this work shows a dielectric constant as low as 1.83 and a dielectric loss of 0.004 at 1 MHz, while maintaining good thermal stability. This combination of ultra-low dielectric constant, low dielectric loss, and high thermal resistance demonstrates the effectiveness of the present molecular design strategy and highlights the potential of these thermosetting benzoxazole systems for high-frequency insulating and electronic packaging applications.

Overall, the dielectric behaviour of these polybenzoxazoles is governed by the combined effects of molecular polarity, free volume, network regularity, and side-group chemistry. Lower polarity, larger free volume, and a more regular cross-linked structure are all favourable for achieving reduced dielectric constant and dielectric loss. These results demonstrate that the present polybenzoxazoles are promising candidates for low-dielectric insulating applications.

**Figure 9:**
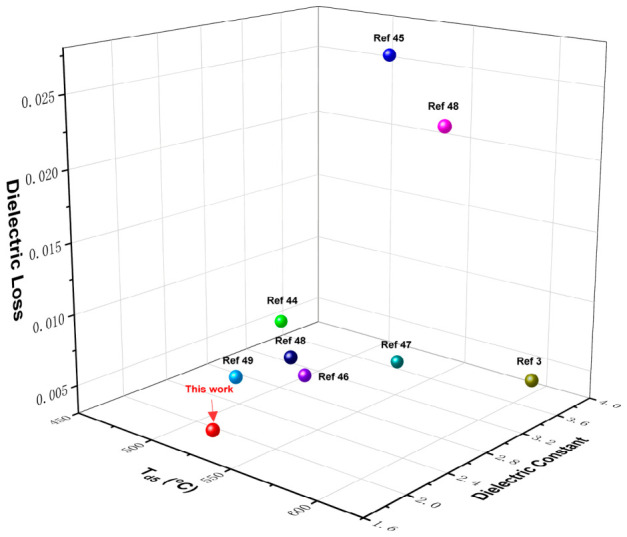
Comparison of T_d5_, dielectric loss and dielectric constant of the material in this work with other high-temperature polymers in the literature.

Figure 9 compares the dielectric properties at 1 MHz and T_d5_ values of poly(S-1) with those of previously reported heat-resistant polymers [3,44,45,46,47,48,49]. The corresponding data are summarized in Table S4. As shown in Figure 9 and Table S4, poly(S-1) exhibits a moderate T_d5_ value compared with the reported systems; however, its dielectric constant is only 1.83, which is the lowest among the compared materials. In addition, poly(S-1) also shows a very low dielectric loss of 0.0039 at 1 MHz. These results indicate that the present thermosetting polybenzoxazole achieves an advantageous balance between thermal stability, ultra-low dielectric constant, and low dielectric loss, demonstrating its potential for high-frequency insulating and electronic packaging applications.

## 4. Conclusions

Two series of thermosetting benzoxazole monomers with distinct molecular architectures were successfully developed to address the long-standing trade-off between processability and high-performance properties in polybenzoxazoles. By combining symmetric hexafluoroisopropylidene-containing structures and asymmetric mono-benzoxazole frameworks with arylethynyl and arylonitrile terminal groups, this study systematically clarifies the effects of terminal-group chemistry and backbone structure on curing behaviour, thermal stability, dielectric properties, solubility, and rheological performance.

Non-isothermal DSC and in situ FTIR analyses reveal that arylethynyl-terminated monomers cure more readily than arylonitrile-terminated analogues, as indicated by their lower apparent activation energies and lower curing-temperature windows. In contrast, arylonitrile-containing systems afford more thermally stable cross-linked networks after curing, owing to the formation of thermally robust nitrogen-containing heterocyclic structures. The introduction of hexafluoroisopropylidene units effectively improves monomer solubility and reduces dielectric constants by lowering molecular polarity and increasing free volume. Among all samples, poly(S-1) exhibits the best low-dielectric performance, with a dielectric constant of 1.83 at 1 MHz, whereas poly(AS-2) shows the highest thermal stability, with a T_d5_ value of 602.2 °C.

Rheological results further demonstrate that several arylonitrile-terminated monomers, especially S-3, S-4, and S-5, possess relatively low melt viscosities and broad processing windows, which indicates strong potential for practical melt processing. Overall, this study demonstrates that simultaneous regulation of terminal reactive groups, substitution patterns, and backbone architecture is an effective molecular design strategy for constructing polybenzoxazole with balanced processability, excellent thermal stability, and favourable dielectric properties. This strategy provides clear guidance for the development of advanced high-temperature insulating materials.

Owing to their low dielectric constants, high thermal stability, and improved solution processability, these polybenzoxazole thermosets are promising candidates for high-temperature dielectric components, microelectronic insulating layers, low-dielectric films, and aerospace-related insulating materials. Future studies may further optimize film formation, curing protocols, and device-level processing to promote their practical use in advanced electronic and high-temperature engineering applications.

## Figures and Tables

**Figure 1:**
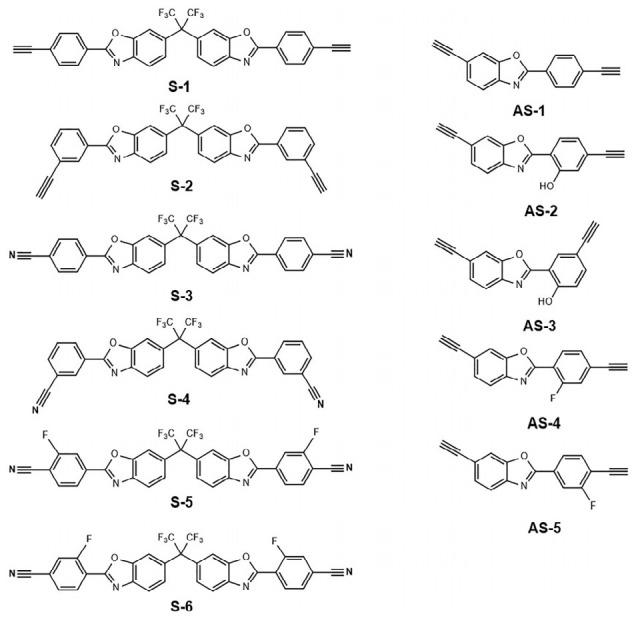
Molecular structures of various benzoxazole monomers.

**Table 1:** Solubility of benzoxazole monomers in common organic solvents.

Solvent	EtOH	Methylbenzene	Acetone	THF	DMF	DMSO
S-1	−	±	+	+	+	+
S-2	−	±	+	+	+	+
S-3	−	±	+	+	+	+
S-4	−	±	+	+	+	+
S-5	−	±	+	+	+	+
S-6	−	±	+	+	+	+
AS-1	−	−	−	±	±	±
AS-2	−	−	−	±	±	±
AS-3	−	−	−	±	±	±
AS-4	−	−	−	±	±	±
AS-5	−	−	−	±	±	±

“+”: soluble; “±”: partially soluble; “−”: insoluble.

**Table 2:** Curing parameters and melt viscosity of S-series monomers.

Monomers	T_m_ (°C)	T_i_ (°C)	T_p_ (°C)	η (Pa‧s)
S-1	206.7	241.0	263.9	>4000
S-2	96.9	197.3	230.9	101.2 (at 150 °C)
S-3	108.5	165.2	201.6	20.6 (at 220 °C)
S-4	167.3	288.9	321.8	21.4 (at 220 °C)
S-5	169.6	289.6	317.6	22.7 (at 220 °C)
S-6	161.1	252.9	307.3	>4000

**Table 3:** Apparent activation energies for the curing reactions of monomers.

Monomer	Ea (kJ/mol)		
	Kissinger	Ozawa	Average
S-1	70.09	74.11	72.10
S-2	68.96	73.54	71.25
S-3	105.9	106.2	106.1
S-5	137.2	139.8	138.5
S-4	114.1	118.0	116.1
S-6	89.31	94.11	91.71
AS-1	52.00	57.43	54.72
AS-2	41.30	46.49	43.90
AS-3	78.92	82.91	80.92
AS-4	58.61	63.60	61.11
AS-5	63.27	68.84	66.06

**Table 4:** Thermogravimetric parameters of polybenzoxazoles.

Polybenzoxazoles	T_d5_ (°C)	Y_c,800_ (%)	Dielectric Constant at 1 MHz	Dielectric Loss at 1 MHz
poly(S-1)	525.7	60.8	1.83	0.0039
poly(S-2)	517.0	67.7	2.43	0.0293
poly(S-3)	537.8	71.2	2.71	0.0154
poly(S-4)	534.8	70.2	2.91	0.0197
poly(S-5)	550.5	72.5	2.59	0.0118
poly(S-6)	554.1	73.2	2.70	0.0486
poly(AS-1)	569.5	75.9	2.68	0.015
poly(AS-2)	602.2	80.0	2.93	0.031
poly(AS-3)	593.5	79.5	2.88	0.029
poly(AS-4)	588.9	77.5	2.55	0.065
poly(AS-5)	582.9	76.8	2.37	0.012

## Data Availability

The original contributions presented in this study are included in the article/Supplementary Material. Further inquiries can be directed to the corresponding author.

## Supplementary Materials

The following supporting information can be downloaded at: https://www.mdpi.com/article/10.3390/polym18111409/s1.

## Abbreviations

The following abbreviations are used in this manuscript:

PBOs	Polybenzoxazoles
^1^H NMR	Proton Nuclear Magnetic Resonance
FTIR	Fourier transform infrared
DSC	Differential scanning calorimetry
TGA	Thermogravimetric analysis
T_d5_	Thermal decomposition temperature at 5% mass loss
T_m_	Melting point
T_i_	Initial curing temperature
T_p_	Peak curing temperatures
Ea	Apparent activation energy
