# Supplementary material for: Balancing Processability and Performance: Benzoxazole Thermosets with Ultra-Low Dielectric Constants and High Thermal Stability
Source: Polymers (Basel). 2026 Jun 5;18(11):1409. doi: 10.3390/polym18111409 (PMC13259294; doi:10.3390/polym18111409)
Supplement: Supplementary file 1 [file polymers-18-01409-s001.zip › polymers-4333129-supplementary.pdf]

Supporting Information

Balancing Processability and Performance: Benzoxazole Thermosets with Ultra-Low Dielectric Constants and High Thermal Stability

Yuchen Ge, Jiaxiong Tian, Qixin Zhuang, Xiaoyun Liu*

Key Laboratory of Specially Functional Polymeric Materials and Related Technology (Ministry of Education), East China University of Science and Technology, Shanghai, 200237, China

*Corresponding Author: Xiaoyun Liu. Email: liuxiaoyun@ecust.edu.cn

1. Synthesis

The synthesis route is shown in Schemes S1-S4. Taking the synthesis of S-2 and AS-1 as an example, the synthesis methods are described.

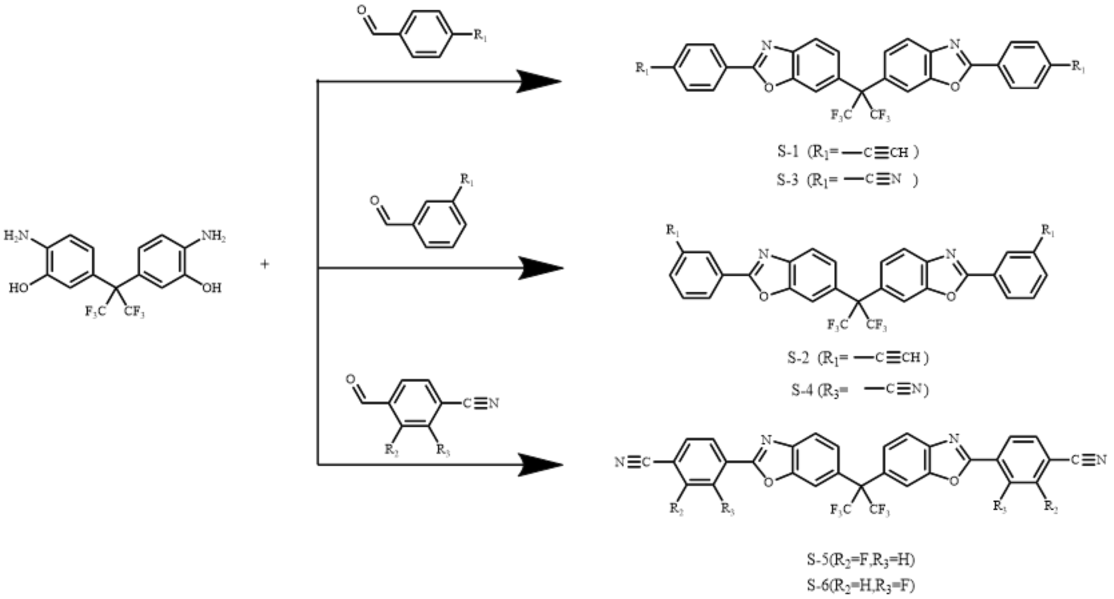

Scheme S1. Synthesis of S-series benzoxazole monomers.

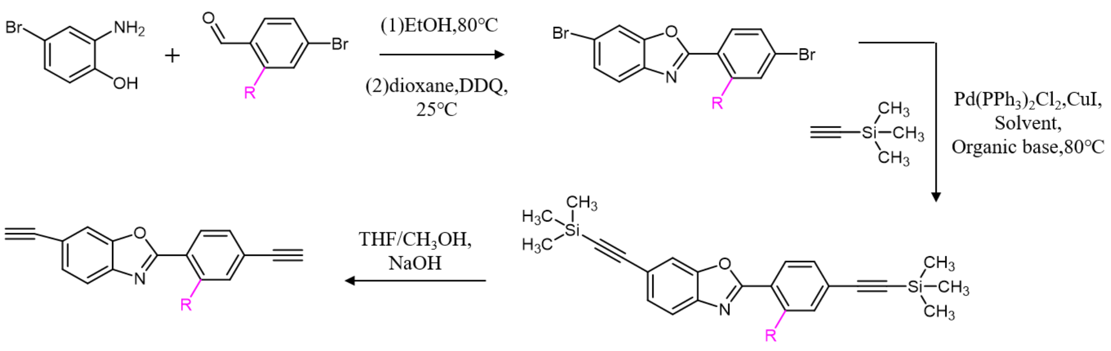

AS-1 (R=H)

AS-2 (R=OH)

AS-4 (R=F)

Scheme S2. Synthesis of AS-1, AS-2, and AS-4 benzoxazole monomers,

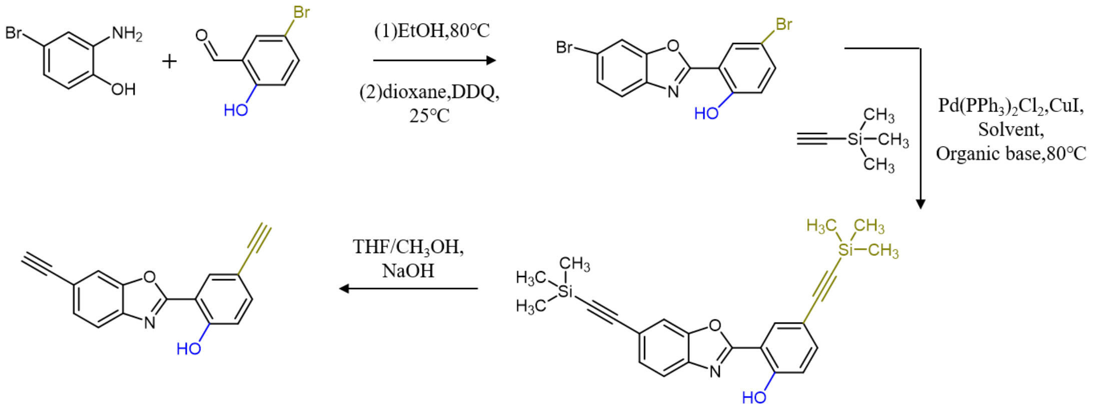

Scheme S3. Synthesis of AS-3 benzoxazole monomer.

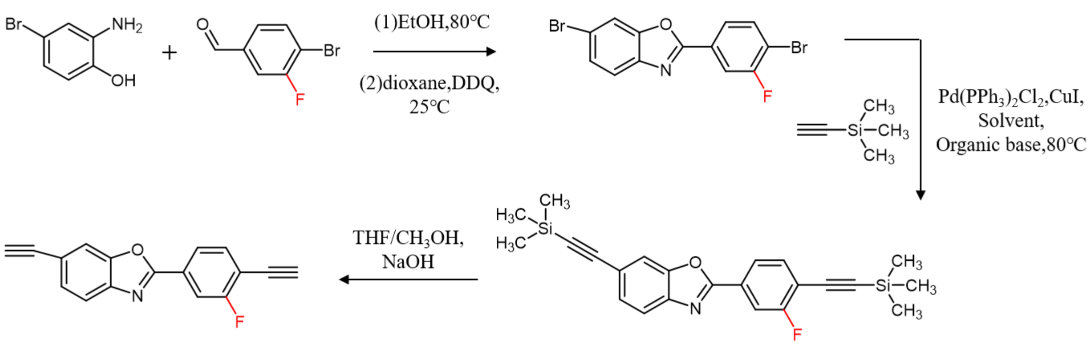

Scheme S4. Synthesis of AS-5 benzoxazole monomer.

1.1. Synthesis of S-2

3-ethynylbenzaldehyde 1.3 g (10 mmol) and 2,2-bis(3-amino-4-hydroxyphenyl) hexafluoropropane 1.83 g (5 mmol) were added to a 250 mL single-necked round-bottom flask, followed by 150 mL of anhydrous ethanol. Subsequently, the reaction was refluxed at 80 °C for 5 h. Then, the reaction was allowed to cool and the solvent was

removed under reduced pressure. Next, 100 mL of 1,4-dioxane and 2,3-Dichloro-5,6-dicyano-1,4-benzoquinone (DDQ) (2.38 g, 10.5 mmol) were added to the flask, and the reaction was carried out at 25 °C for 24 h. Subsequently, the system was filtered, and the filtrate was concentrated under reduced pressure. 900 mL of saturated $NaHCO_3$ solution was prepared in a beaker, and after removing approximately half of the solvent under reduced pressure, the remaining filtrate was poured into the prepared saturated $NaHCO_3$ solution. A large amount of faint yellow precipitate emerged. When the product was completely precipitated, the system was filtered, and the precipitate was dried in a vacuum at 75 °C. The crude product was washed several times with anhydrous ethanol to produce the purified S-2.

The synthetic procedure for S-1 was identical to that of S-2, except for the aldehyde precursor used; all other treatment procedures were the same. The intermediate obtained was an off-white powder, and the final product was a pale-yellow powder with a yield of approximately 90%. The synthetic procedures for S-3, S-4, S-5, and S-6 were also the same as described above, differing only in the aldehyde precursor, while all other treatment procedures remained unchanged. All intermediates were obtained as yellow powders. The final products, S-3, S-4, and S-5, were obtained as yellow powders with yields of approximately 89%, 87%, and 92%, respectively, whereas S-6 was obtained as a brick-red powder with a yield of approximately 93%.

1.2. Synthesis of AS-1

(1) 4-Bromo-2-aminophenol (10 mmol, 1.88 g) and 4-bromobenzaldehyde (10 mmol, 1.85 g) were added to a 250 mL single-neck round-bottom flask, followed by 120-150 mL of anhydrous ethanol to dissolve the reactants completely. The flask was equipped with a reflux condenser and a magnetic stirrer, and the mixture was heated at 80 °C for 5 h. After the reaction was completed, the mixture was cooled to room temperature, and the solvent was removed under reduced pressure to afford a dark brown solid. The crude product was then redissolved in an appropriate amount of dioxane to form a clear solution, after which DDQ (2.38 g, 10.5 mmol) was added, giving a dark-colored mixture. The resulting mixture was stirred at 25 °C for 24 h and then filtered to remove the insoluble DDQ byproducts. Most of the solvent in the filtrate was removed under reduced pressure, and the remaining solution was poured into 800-1000 mL of saturated $NaHCO_3$ aqueous solution to induce precipitation. After standing for a period of time, the precipitate was collected by vacuum filtration to obtain the crude product. The solid was dried in an oven, washed with an appropriate amount of

anhydrous ethanol to remove unreacted starting materials and intermediates, and then dried at 75 °C to afford Br-EZ as a yellow-brown powder (3.0 g, 85%).

(2) A 250 mL three-neck round-bottom flask equipped with a condenser and a stirring device was purged with argon for 30 min. Br-EZ (5 mmol, 1.76 g), bis(triphenylphosphine)palladium(II) dichloride (0.05 mmol, 0.176 g), and cuprous iodide (0.1 mmol, 0.095 g) were then introduced into the flask. Subsequently, dry DMF (80 mL) and diisopropylethylamine (10 equiv) were added, and the reaction system was further degassed by three vacuum-argon refill cycles to remove residual oxygen while maintaining an argon atmosphere. After the reaction temperature reached the desired value, trimethylsilylacetylene (15 mmol, 1.62 g) was added dropwise to the sealed system using a syringe. The apparatus was then tightly sealed, and the reaction mixture was stirred at 80 °C for 24 h under reflux. After cooling, the reaction mixture was poured into deionized water, and the product was extracted with ethyl acetate. The organic phase was separated, dried over anhydrous $MgSO_4$, and filtered. The solvent was removed under reduced pressure, and the resulting solid was dried under vacuum at 75 °C to give Br-Si as a dark brown powder.

(3) Deprotection of the trimethylsilyl (TMS) group: Br-Si was placed in a flat-bottom flask or beaker and dissolved in THF under stirring. An equal volume of CH_3OH was then added, affording a dark brown solution. Subsequently, 1 mol/L NaOH aqueous solution was added dropwise, and the mixture was stirred at room temperature for 45 min. After completion of the reaction, the product was collected by filtration and dried under vacuum at 75 °C to afford AS-1 as a yellow-brown powder (0.77 g, 85%).

The first and third synthetic steps for AS-2, AS-3, AS-4, and AS-5 were the same as those for AS-1, whereas the second step was carried out using a triethylamine system.

2. Curing schedules

All benzoxazole monomers were placed in a tube furnace under a nitrogen atmosphere and subjected to stepwise curing at a heating rate of 5 °C min^{-1} to obtain the corresponding polybenzoxazoles. Curing schedules are as follows:

(1) **S-2, S-3, S-4, S-5, S-6, AS-1**: 160 °C/1 h, 200 °C/1 h, 230 °C/2 h, 250 °C/2 h, 300 °C/1 h, and 350 °C/1 h.

(2) **S-1**: 100 °C/1 h, 150 °C/1 h, 200 °C/2 h, 230 °C/2 h, 300 °C/1 h, and 350 °C/1 h.

(3) **AS-2, AS-3, AS-4, AS-5**: 120 °C/1 h, 160 °C/1 h, 200 °C/1 h, 250 °C/2 h, 300 °C/2 h, 350 °C/1 h, and 380 °C/1 h.

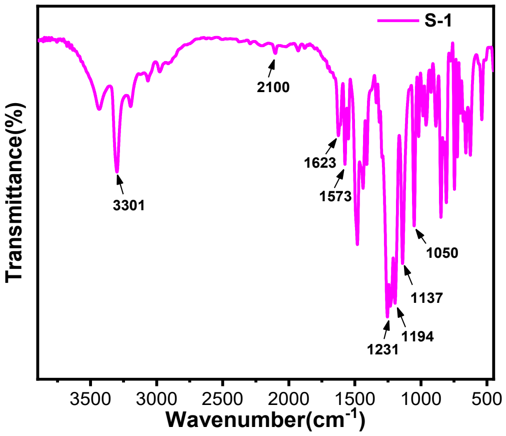

Figure S1. FTIR spectra of S-1.

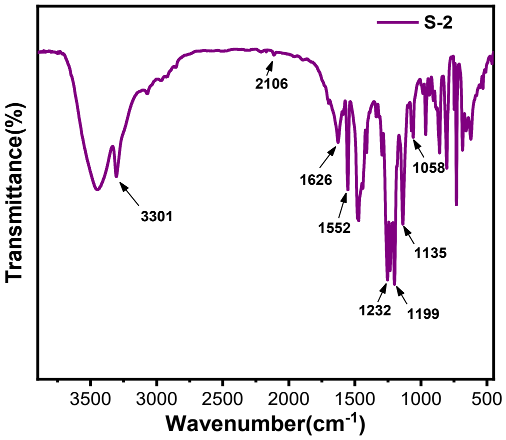

Figure S2. FTIR spectra of S-2.

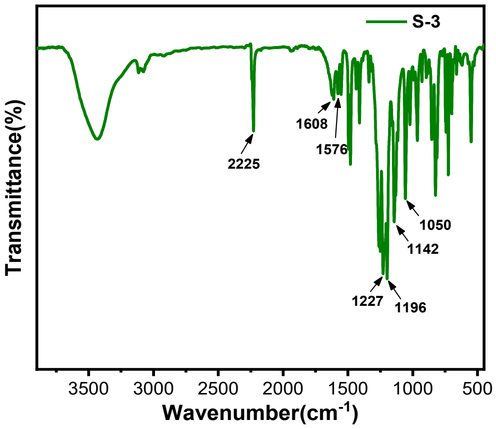

Figure S3. FTIR spectra of S-3.

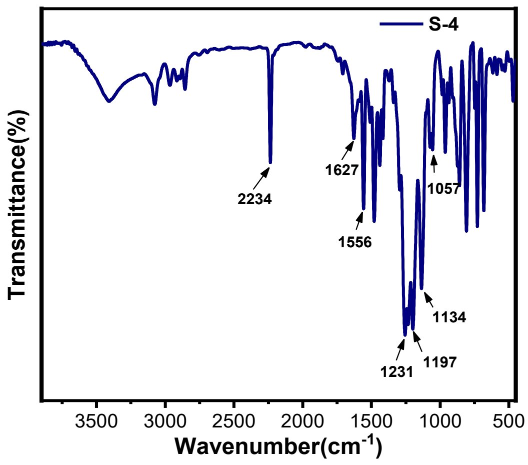

Figure S4. FTIR spectra of S-4.

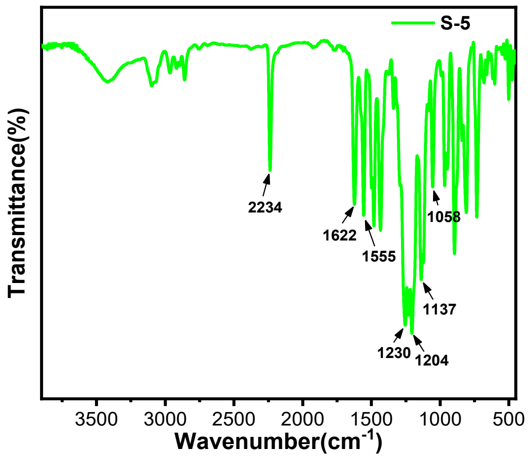

Figure S5. FTIR spectra of S-5.

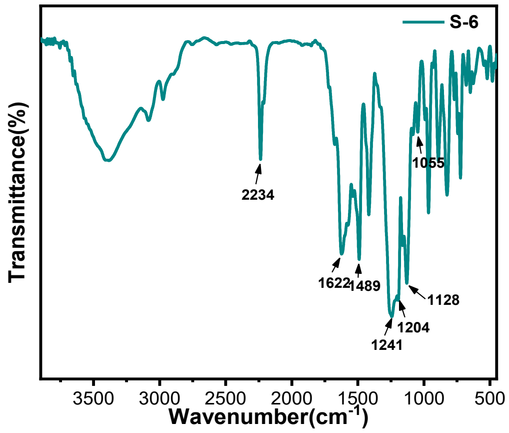

Figure S6. FTIR spectra of S-6.

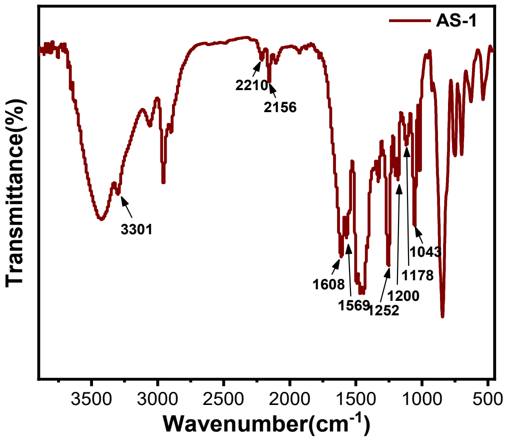

Figure S7. FTIR spectra of AS-1.

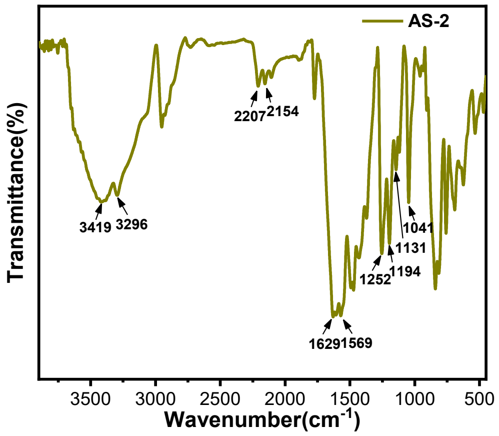

Figure S8. FTIR spectra of AS-2.

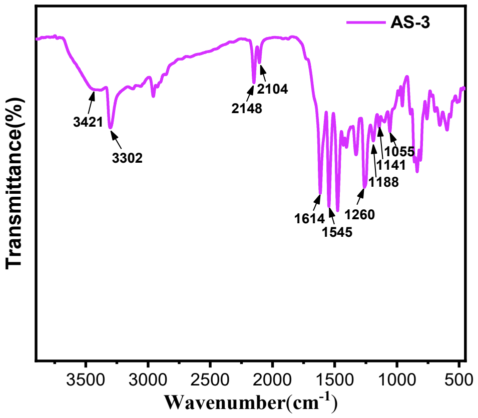

Figure S9. FTIR spectra of AS-3.

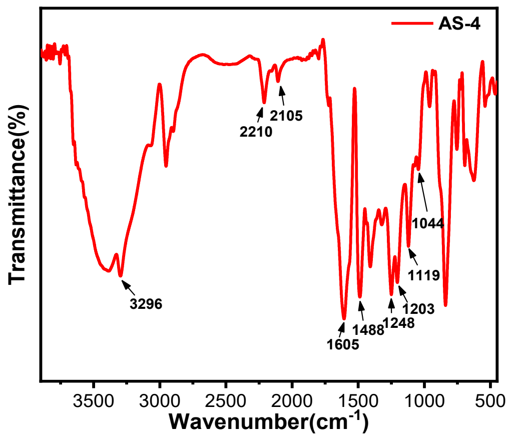

Figure S10. FTIR spectra of AS-4.

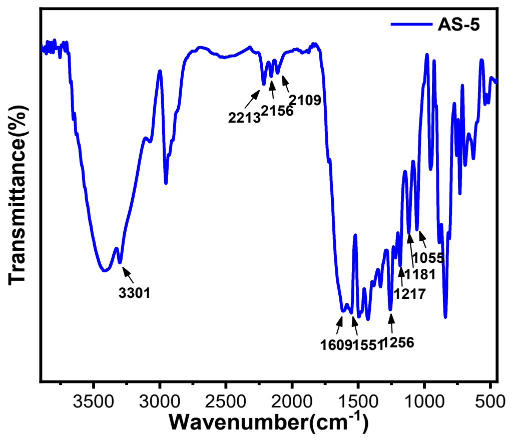

Figure S11. FTIR spectra of AS-5.

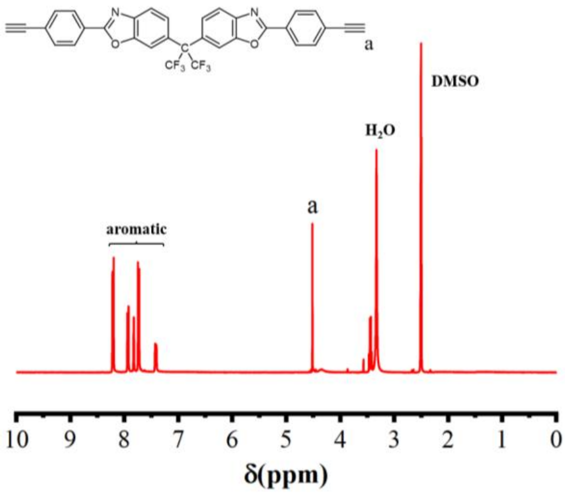

Figure S12. ^{1}H-NMR spectra of S-1.

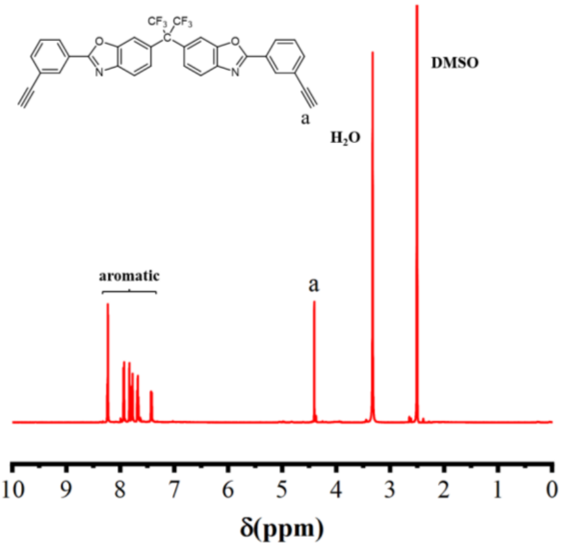

Figure S13. ^{1}H-NMR spectra of S-2.

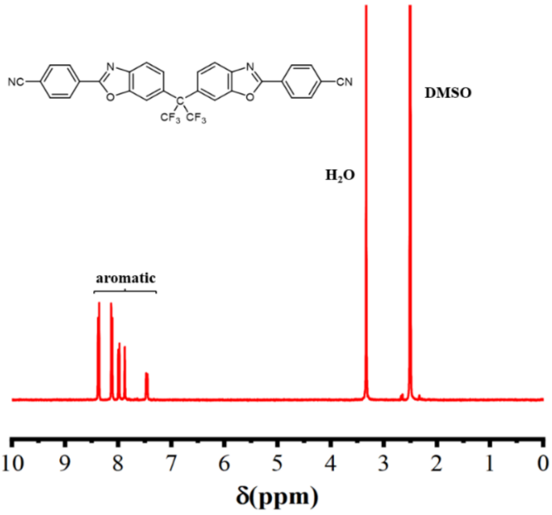

Figure S14. ^{1}H-NMR spectra of S-3.

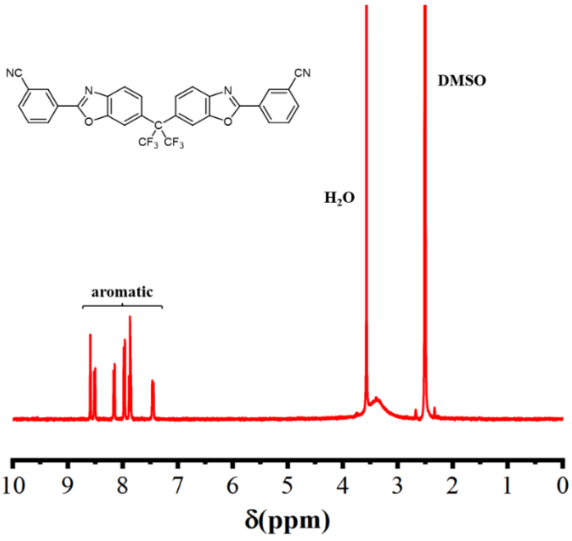

Figure S15. ^{1}H-NMR spectra of S-4.

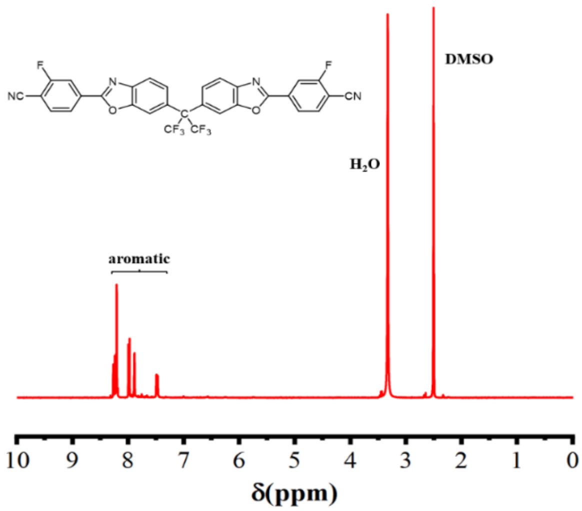

Figure S16. ^{1}H-NMR spectra of S-5.

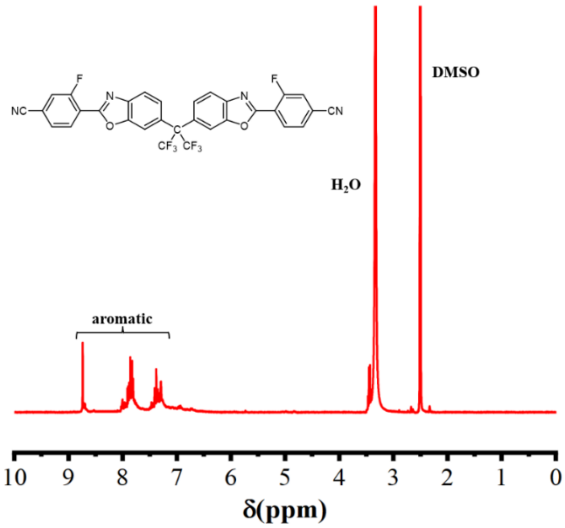

Figure S17. ^{1}H-NMR spectra of S-6.

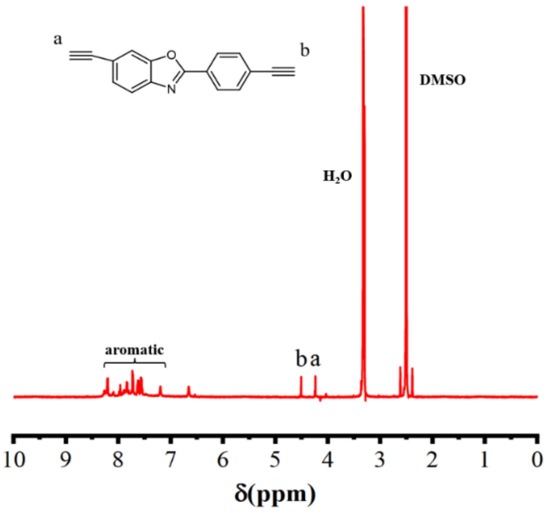

Figure S18. ^{1}H-NMR spectra of AS-1.

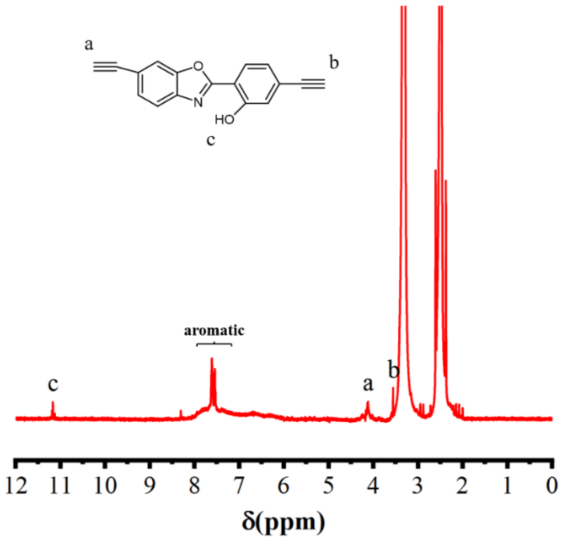

Figure S19. ^{1}H-NMR spectra of AS-2.

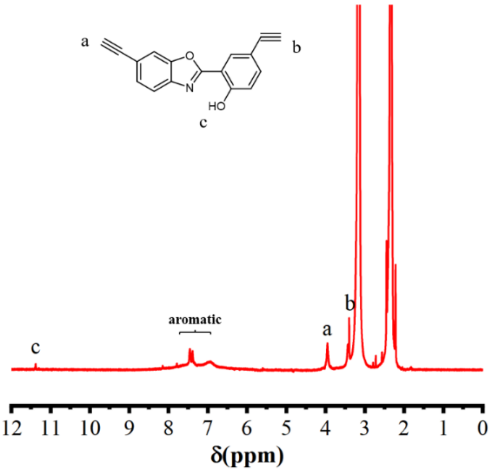

Figure S20. ^{1}H-NMR spectra of AS-3.

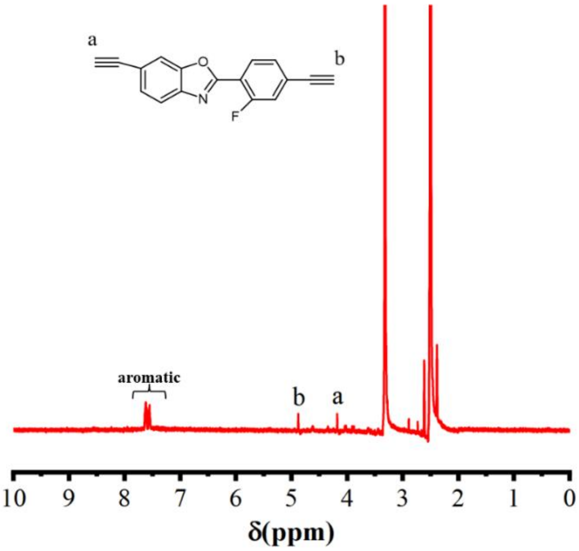

Figure S21. ^{1}H-NMR spectra of AS-4.

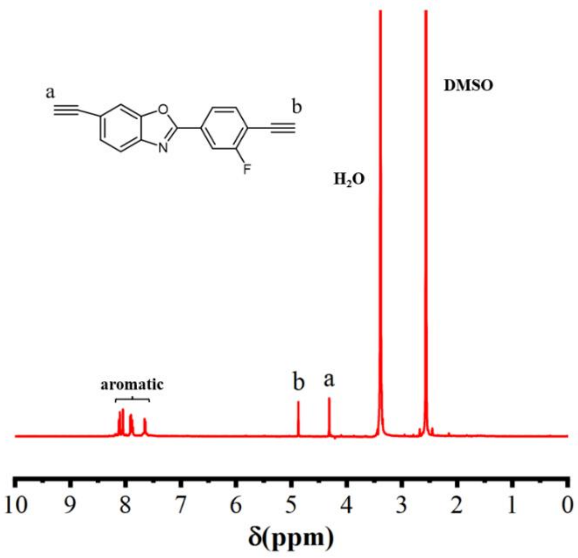

Figure S22. ^{1}H-NMR spectra of AS-5.

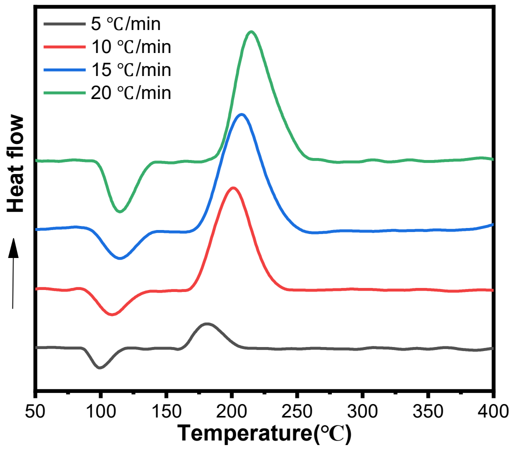

Figure S23. DSC curves of S-1 at different heating rates.

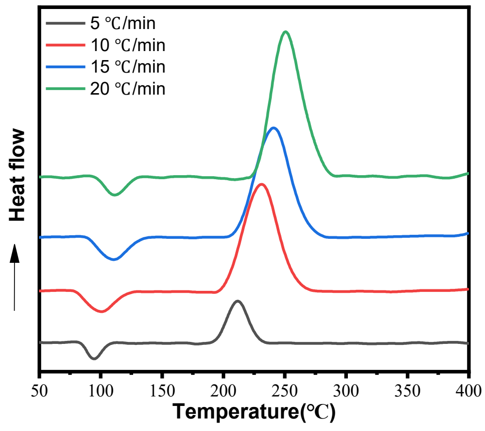

Figure S24. DSC curves of S-2 at different heating rates.

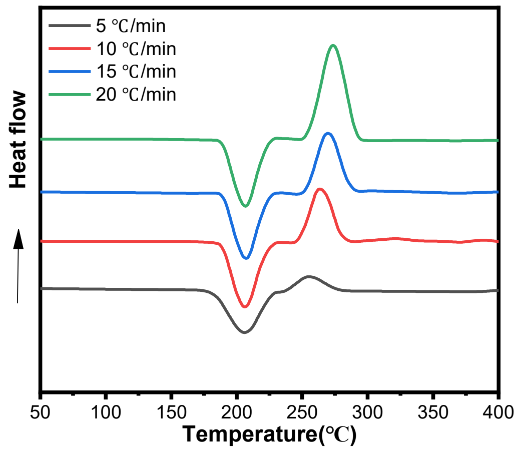

Figure S25. DSC curves of S-3 at different heating rates.

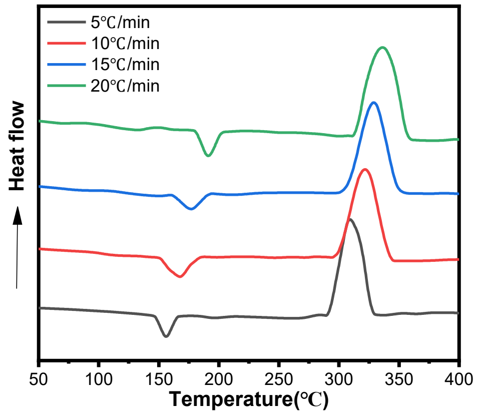

Figure S26. DSC curves of S-4 at different heating rates.

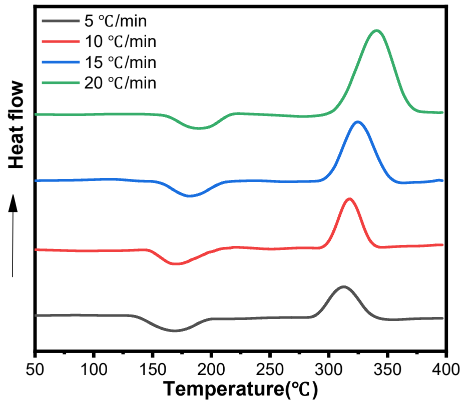

Figure S27. DSC curves of S-5 at different heating rates.

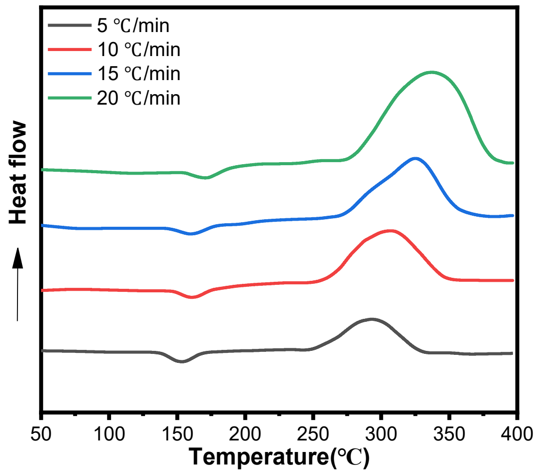

Figure S28. DSC curves of S-6 at different heating rates.

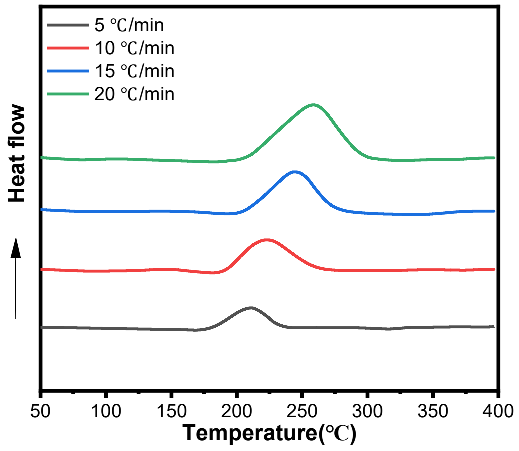

Figure S29. DSC curves of AS-1 at different heating rates.

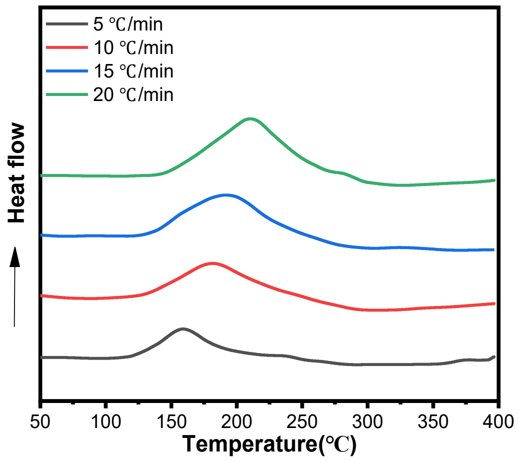

Figure S30. DSC curves of AS-2 at different heating rates.

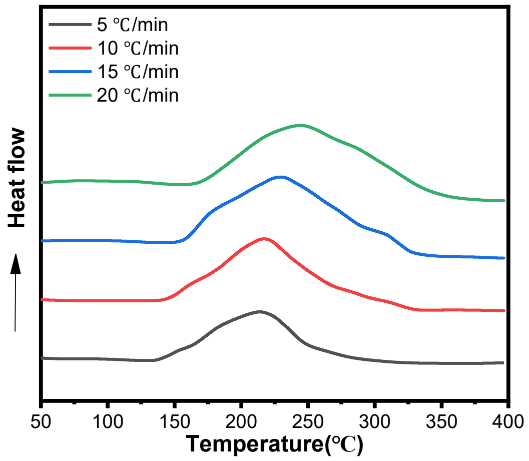

Figure S31. DSC curves of AS-3 at different heating rates.

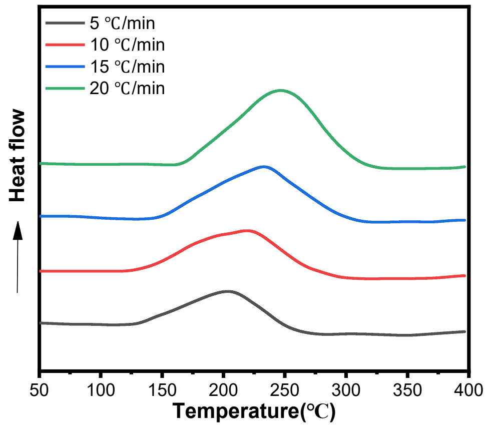

Figure S32. DSC curves of AS-4 at different heating rates.

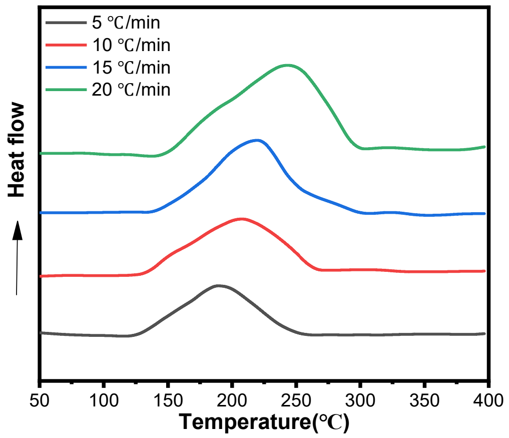

Figure S33. DSC curves of AS-5 at different heating rates.

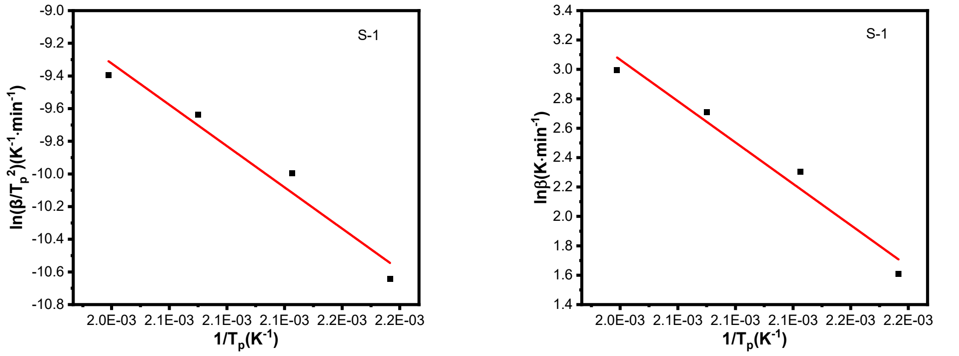

Figure S34. Kissinger (left) and Ozawa (right) plots of S-1.

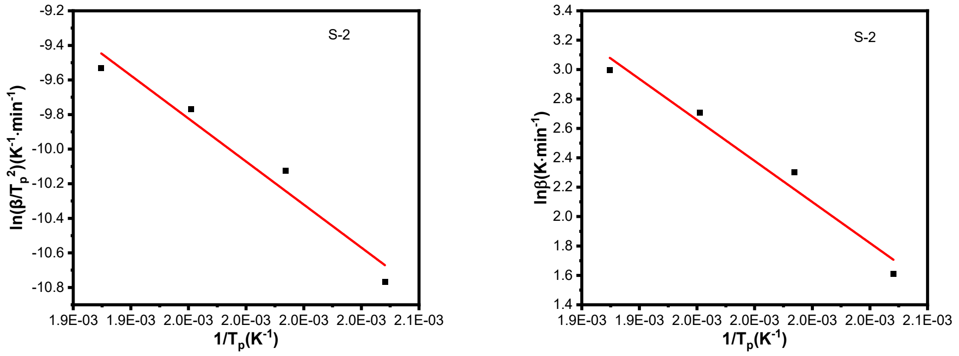

Figure S35. Kissinger (left) and Ozawa (right) plots of S-2.

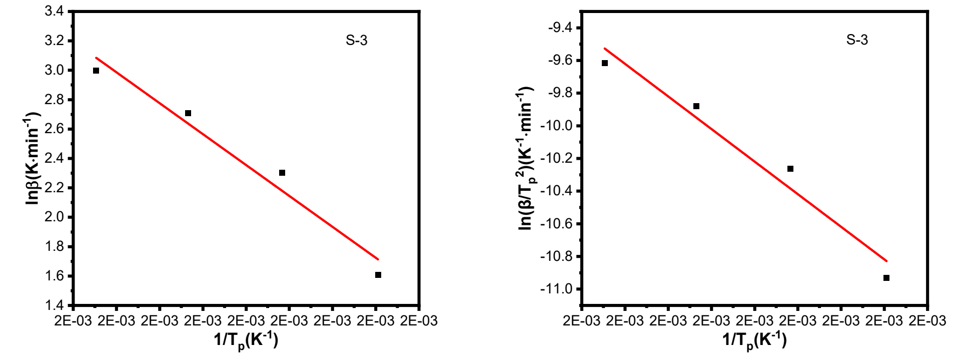

Figure S36. Kissinger (left) and Ozawa (right) plots of S-3.

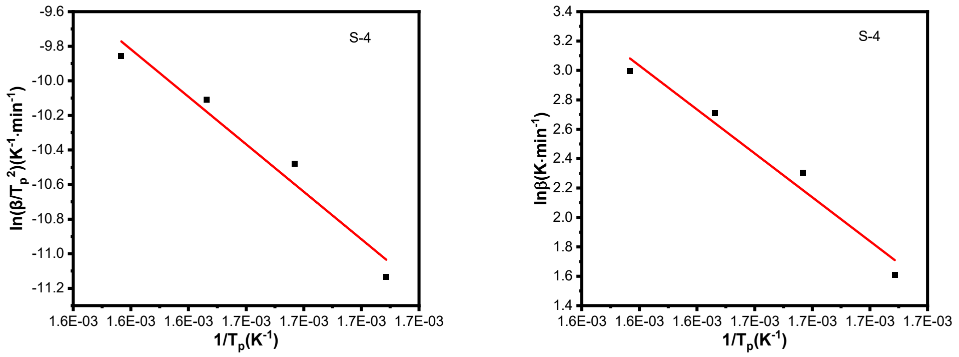

Figure S37. Kissinger (left) and Ozawa (right) plots of S-4.

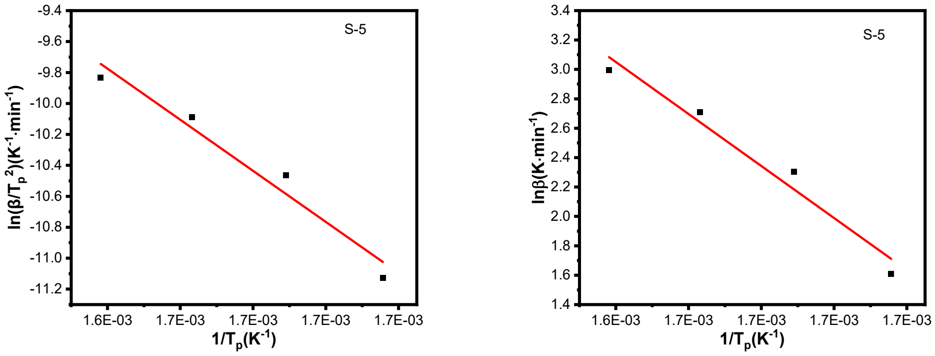

Figure S38. Kissinger (left) and Ozawa (right) plots of S-5.

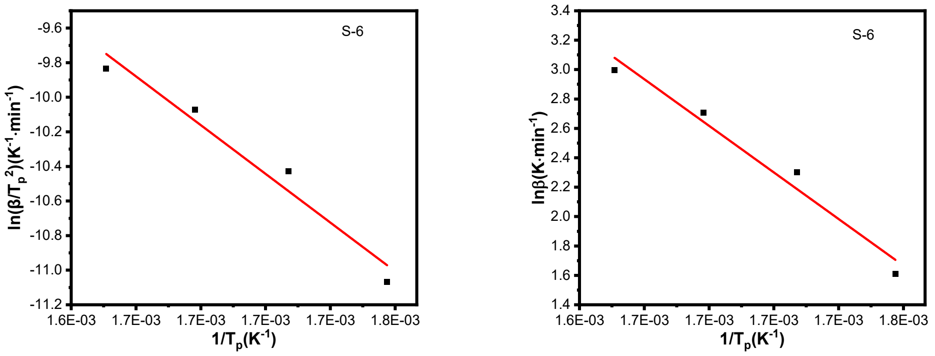

Figure S39. Kissinger (left) and Ozawa (right) plots of S-6.

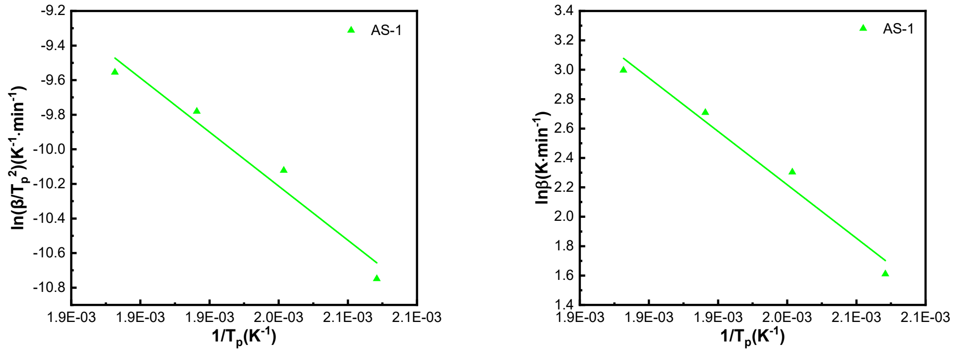

Fig.S40. Kissinger (left) and Ozawa (right) plots of AS-1.

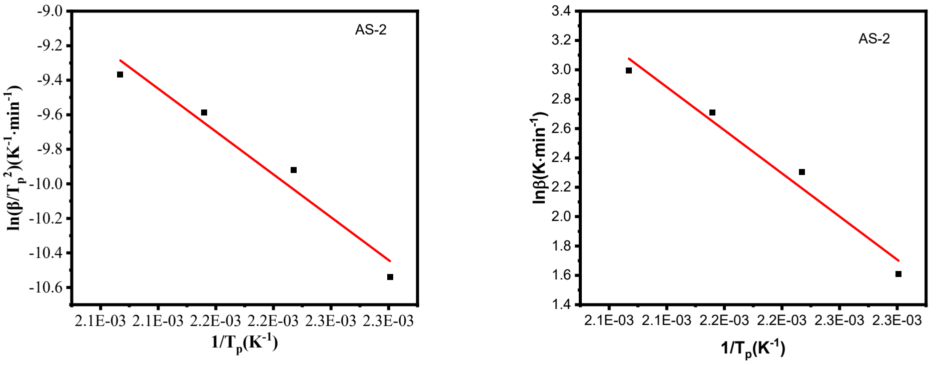

Figure S41. Kissinger (left) and Ozawa (right) plots of AS-2.

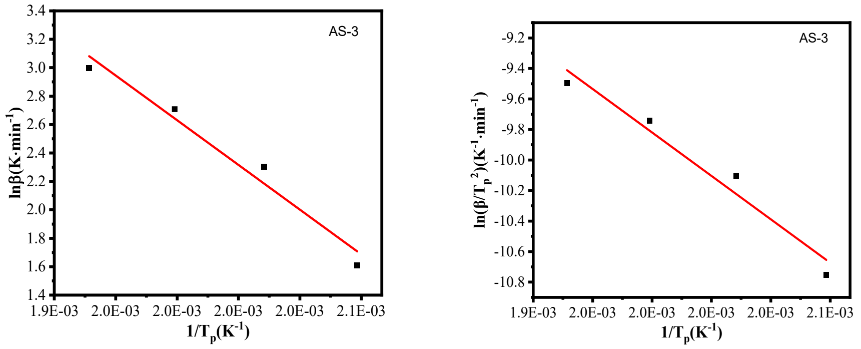

Figure S42. Kissinger (left) and Ozawa (right) plots of AS-3.

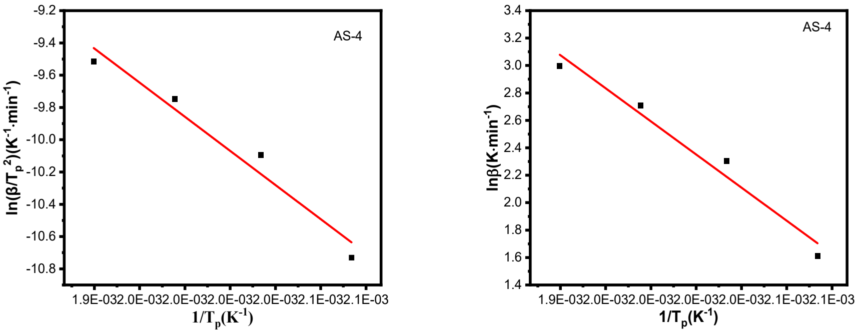

Fig.S43. Kissinger (left) and Ozawa (right) plots of AS-4.

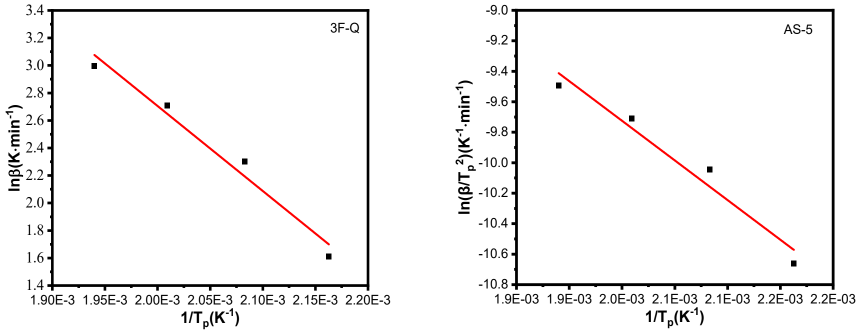

Fig.S44. Kissinger (left) and Ozawa (right) plots of AS-5.

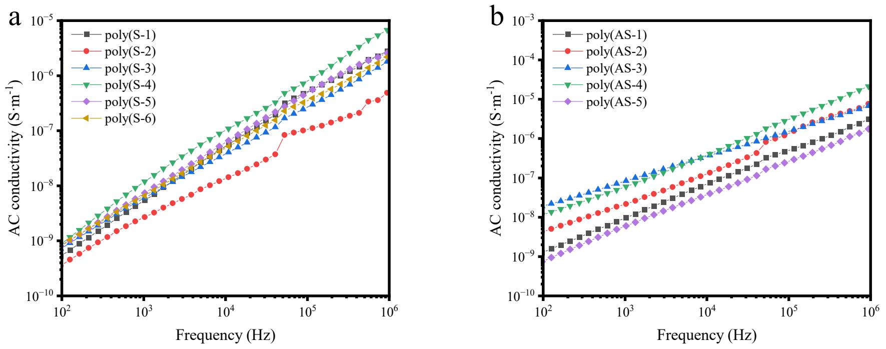

Figure S45. AC conductivity of cured polybenzoxazoles: (a) S-series; (b) AS-series.

Table S1. Assignments of characteristic FTIR absorption bands and ^{1}H NMR signals of the synthesized benzoxazole monomers.

Monomer	Characteristic FTIR absorption bands	Main ^{1}H NMR signals	Structural assignment
S-1, S-2	≡C–H stretching at ca. 3300 cm^{-1}; C≡C stretching at ca. 2100 cm^{-1}; aromatic/benzoxazole ring vibrations at 1600-1450 cm^{-1}; C–F vibrations from hexafluoroisopropylidene units at 1250-1050 cm^{-1}	aromatic protons at ca. 7.0-8.5 ppm; terminal ethynyl protons at ca. 4.0-4.3 ppm	Formation of benzoxazole rings and retention of terminal ethynyl groups
S-3, S-4	C≡N stretching at ca. 2220-2240 cm^{-1}; aromatic/benzoxazole ring vibrations at 1600-1450 cm^{-1}; C–F vibrations from hexafluoroisopropylidene units at 1250-1050 cm^{-1}	aromatic protons at ca. 7.0-8.5 ppm	Formation of benzoxazole rings and introduction of terminal cyano groups
S-5, S-6	C≡N stretching at ca. 2220-2240 cm^{-1}; aromatic/benzoxazole ring vibrations at 1600-1450 cm^{-1}; C–F vibrations from hexafluoroisopropylidene/fluorinated substituents at 1250-1050 cm^{-1}	aromatic protons at ca. 7.0-8.5 ppm	Formation of benzoxazole rings and introduction of cyano/fluorinated substituents
AS-1	≡C–H stretching at ca. 3300 cm^{-1}; C≡C stretching at ca. 2100 cm^{-1}; aromatic/benzoxazole ring vibrations at 1600-1450 cm^{-1}	aromatic protons at ca. 7.0-8.5 ppm; terminal ethynyl protons at ca. 4.0-4.3 ppm	Formation of asymmetric mono-benzoxazole structure with terminal ethynyl groups
AS-2, AS-3	O–H stretching at ca. 3200–3600 cm^{-1}; ≡C–H stretching at ca. 3300 cm^{-1}; C≡C stretching at ca. 2100 cm^{-1}; aromatic/benzoxazole ring vibrations at 1600–1450 cm^{-1}	hydroxyl proton at ca. 10-12 ppm; aromatic protons at ca. 7.0-8.5 ppm; terminal ethynyl protons at ca. 4.0-4.3 ppm	Formation of hydroxyl-containing asymmetric benzoxazole monomers with terminal ethynyl groups
AS-4, AS-5	≡C–H stretching at ca. 3300 cm^{-1}; C≡C stretching at ca. 2100 cm^{-1}; aromatic/benzoxazole ring vibrations at 1600–1450 cm^{-1}; C–F stretching at ca. 1250–1050 cm^{-1}	aromatic protons at ca. 7.0-8.5 ppm; terminal ethynyl protons at ca. 4.0-4.3 ppm	Formation of fluorinated asymmetric benzoxazole monomers with terminal ethynyl groups

Table S2. Summary of fitting data of Ti, Tp, and Tf of S-series monomers.

**Resins**	**Heating rate(°C/min)**	**T_i(°C)**	**T_p(°C)**	**T_f(°C)**
S-1	5	159.1	184.5	217.8
	10	165.2	195.5	236.6
	15	171.3	206.5	255.4
	20	177.4	217.4	274.2
S-2	5	187.9	214.1	242.8
	10	197.3	226.7	260.6
	15	206.7	239.3	278.4
	20	216.1	251.9	296.2
S-3	5	235.0	255.8	284.0
	10	241.0	262.1	289.0
	15	247.0	268.4	294.0
	20	253.0	274.7	299.0
S-4	5	281.2	312.1	346.1
	10	288.9	323.2	357.2
	15	296.6	334.3	368.3
	20	304.3	345.4	379.4
S-5	5	284.8	309.7	341.3
	10	289.6	318.9	354.1
	15	294.4	328.1	366.9
	20	299.2	337.3	379.7
S-6	5	243.2	293.0	337.4
	10	252.9	308.0	355.3
	15	262.6	323.0	373.2
	20	272.3	338.0	391.1

Table S3. Summary of Ti, Tp, and Tf fitting data Results of AS-series monomers.

**Resins**	**Heating rate(°C /min)**	**Ti(°C)**	**Tp(°C)**	**Tf(°C)**
AS-1	5	175.8	209.7	243.7
	10	186.1	225.9	264.4
	15	196.4	242.1	285.1
	20	206.7	258.3	305.8
AS-2	5	113.7	161.4	224.8
	10	122.4	177.8	241.6
	15	131.1	194.2	258.4
	20	139.8	210.6	275.2
AS-3	5	131.2	210.4	315.5
	10	141.4	221.3	330.5
	15	151.6	232.2	345.5
	20	161.8	243.1	360.5
AS-4	5	115.6	205.2	286
	10	129.7	219.4	301.5
	15	143.8	233.6	317.0
	20	157.9	247.8	332.5
AS-5	5	118.6	189.2	256.9
	10	126.7	206.9	272.3
	15	134.8	224.6	287.7
	20	142.9	242.3	303.1

Table S4. Comparison of the thermal and dielectric properties of the resins prepared in this work with other high temperature resistant resins in the literature.

Polymer/composite	Td_5 (°C)	Dielectric constant at 1 MHz	Dielectric loss at 1 MHz	ref
poly(DDM-BMI)	475	3.2	0.006	44
Neat PI	521	3.6	0.026	45
FPBO	523	2.7	0.005	46
PI	534	3.5	0.004	47
oda-pmda	582	3.1	0.023	48
oda-pmda with 10% PM	527	2.5	0.007	48
PI/K-HGM-8%	512	2.2	0.006	49
poly(2F-JQ)	610	3.6	0.005	3
poly(S-1)	525	1.8	0.004	This work

References

[3]. Liu S L, Yang R, Zhuang Q X, et al. Thermosetting benzoxazoles with endo-alkynyl groups: Excellent thermal stability and low dielectric loss. Polymer, 2024, 307: 127318

[44]. Zhang K, Hao B R, Ishida H. Synthesis of a smart bisbenzoxazine with combined advantages of bismaleimide and benzoxazine resins and its unexpected formation of very high performance cross-linked polybenzoxazole. Polymer, 2021, 223(2021): 123703.

[45]. Qian G T, Hu M J, Zhang S Y, et al. Synthesis of Superheat-Resistant Polyimides with Enhanced Dielectric Constant by Introduction of Cu(II)-Coordination. Polymers, 2020, 12(2): 442.

[46]. Yu Z H, Wu S H, Li C C, et al. Ultra-low dielectric constant fluorinated graphene/ polybenzoxazole composite films with excellent thermal stabilities and mechanical properties. Composites Part A-Applied Science and Manufacturing, 2021, 145(2021): 106387.

[47]. Cai L X, Wu J M, Qin H M, et al. High-temperature resistant polyimide-based sandwich-structured dielectric nanocomposite films with enhanced energy density and efficiency. Journal of Applied Polymer Science, 2021, 138(43): e51268.

[48]. Qiu G R, Ma W S, Wu L. Low dielectric constant polyimide mixtures fabricated by polyimide matrix and polyimide microsphere fillers. Polymer International, 2020, 69(5): 485-491.

[49]. Cao X W, Wen J W, Song L H, et al. Polyimide hollow glass microspheres composite films with low dielectric constant and excellent thermal performance. Journal of Applied Polymer Science, 2021, 138(25): e50600.
